# LMNA R482L mutation causes impairments in C2C12 myoblasts subpopulations, alterations in metabolic reprogramming during differentiation, and oxidative stress

**DOI:** 10.1038/s41598-025-88219-6

**Published:** 2025-02-13

**Authors:** Oksana A. Ivanova, Alexander V. Predeus, Margarita Y. Sorokina, Elena V. Ignatieva, Danila E. Bobkov, Kseniia S. Sukhareva, Anna A. Kostareva, Renata I. Dmitrieva

**Affiliations:** 1 Research Centre for Personalized Medicine, Almazov National Medical Research Centre, 2 Akkuratova St., Saint Petersburg, 197341 Russia; 2 Institute of Molecular Biology and Genetics, Almazov National Medical Research Centre, 2 Akkuratova St., Saint Petersburg, 197341 Russia; 3 Bioinformatics Institute, 2A Kantemirovskaya St., Saint Petersburg, 194100 Russia; 4 Institute of Cytology, Russian Academy of Sciences, 4 Tikhoretsky Av., Saint Petersburg, 194064 Russia

**Keywords:** Mechanisms of disease, Stem-cell differentiation, Muscle stem cells, Neuromuscular disease, Transcriptomics

## Abstract

LMNA mutations causing classical familial partial lipodystrophy of Dunnigan type (FPLD2) usually affect residue R482. FPLD is a severe metabolic disorder that often leads to cardiovascular and skeletal muscle complications. How LMNA mutations affect the functional properties of skeletal muscles is still not well understood. In the present project, we investigated the LMNA-R482L mutation-specific alterations in a transgenic mouse C2C12 cell line of myoblasts. Using single-cell RNA sequencing we have studied transcriptional diversity of cultured in vitro C2C12 cells. The LMNA-R482L mutation induces changes in C2C12 cluster composition and increases the expression of genes related to connective tissue development, oxidative stress, stress defense, and autophagy in a population-specific manner. Bulk RNA-seq confirmed these results and revealed the dysregulation of carbohydrate metabolism in differentiated R482L myotubes that was supported by ATP production profile evaluation. The measurement of reactive oxygen species (ROS) levels and glutathione accumulation in myoblasts and myotubes indicates R482L mutation-related dysregulation in mechanisms that control ROS production and scavenging through antioxidant glutathione system. The increased accumulation of autophagy-related structures in R482L myoblasts was also shown. Overall, our experiments showed a connection between the redox status and metabolic alterations with skeletal muscle pathological phenotypes in cells bearing pathogenic LMNA mutation.

## Introduction

LMNA mutations causing classical familial partial lipodystrophy of Dunnigan type (FPLD2) usually affect residue R482. FPLD2 patients may also develop muscular dystrophy^1^, muscle fibers hypertrophy^2^, dilated cardiomyopathy^3^, proteinuric nephropathy^4^, hypertriglyceridemia, and insulin resistance that may progress to type 2 diabetes mellitus (T2DM)^5–7^. Muscle hypertrophy of the lower extremities, along with muscular pain and exercise intolerance, is a common feature of FPLD syndromes, including FPLD2^2,5,8^. However, in FPLD2, increased muscle volume negatively correlates with insulin sensitivity and myofiber metabolic efficiency^5,8^. It is hypothesized that the general metabolic alterations in FPLD2 could result from a combination of intrinsic defects in muscle metabolism and an impaired ability of adipose tissue to store lipids^5^.

Multiple mechanisms for laminopathies have been proposed, including impaired structural nuclear and cellular integrity resulting in increased fragility, aberrant gene expression, defective DNA repair, and prelamin A toxicity^9^. However, the exact mutation-specific molecular mechanisms leading to functional alterations in skeletal muscle are still under debate.

There is evidence in the literature that oxidative stress and reactive oxygen species (ROS) accumulation in adipocytes and fibroblasts, or increased plasma levels of ROS might contribute to the pathogenesis of laminopathies characterized by lipodystrophy and premature aging disorders^10–12^. Muscle cells are very sensitive to alterations in redox homeostasis^13^, therefore, inherited skeletal muscle and cardiac diseases are often associated with imbalances in ROS accumulation and elimination^10,13–15^. Furthermore, myoblasts rely on metabolic reprogramming during the transition from proliferation state to differentiation (reviewed by Ryall in^16^). This reprogramming is accompanied by increased mitochondrial biogenesis and OXPHOS-related ATP production and subsequent increase in ROS levels^13,16^. Under physiological concentration in differentiating myoblasts, ROS regulate proper signaling necessary for myotube development and activate important metabolic genes such as PGC-1α, nuclear respiratory factor NRF1/2, and estrogen-related receptor-α (ERR-α)^17^. However, excessive levels of ROS and insufficient stress defense activation are known to damage mitochondrial metabolism and reduce viability of muscle cells and differentiation efficiency^13,17^. Previously, we have shown that C2C12 mouse myoblasts genetically modified to express human LMNA (hLMNA) gene bearing R482L mutation exhibited alterations in coordination of cell cycle dynamics and muscle differentiation, mitochondrial uncoupling, and a decrease in glycolytic activity in skeletal muscle myofiber^18–20^. At the same time, the exact regulatory connections between the redox status of skeletal muscle and aberrant differentiation in FPLD2 remain to be found.

Importantly, skeletal muscle myofiber is multinucleated, and each nucleus appeared to have a distinct expression pattern according to a fiber type, or nuclei location alongside myotendinous junction (MTJ) or neuromuscular junction (NMJ) (reviewed in^21^). The heterogeneity of myofiber’s nuclei apparently comes from the expressional heterogeneity of differentiating myoblasts^21–23^ and has been shown for C2C12 line differentiated for 72 h using single-nuclei Split-seq^24^, and for C2C12 cells induced to differentiate while being cultured under various mechanical stresses^25^. However, for classical C2C12 myoblasts cell line the data about the heterogeneity is very limited, and still little is known about the role of environment and regulatory cross-talk between distinct populations of myoblasts during skeletal muscle cell functionalization, as well as about the role of specific subpopulations in laminopathy pathogenesis.

**Fig. 1:**
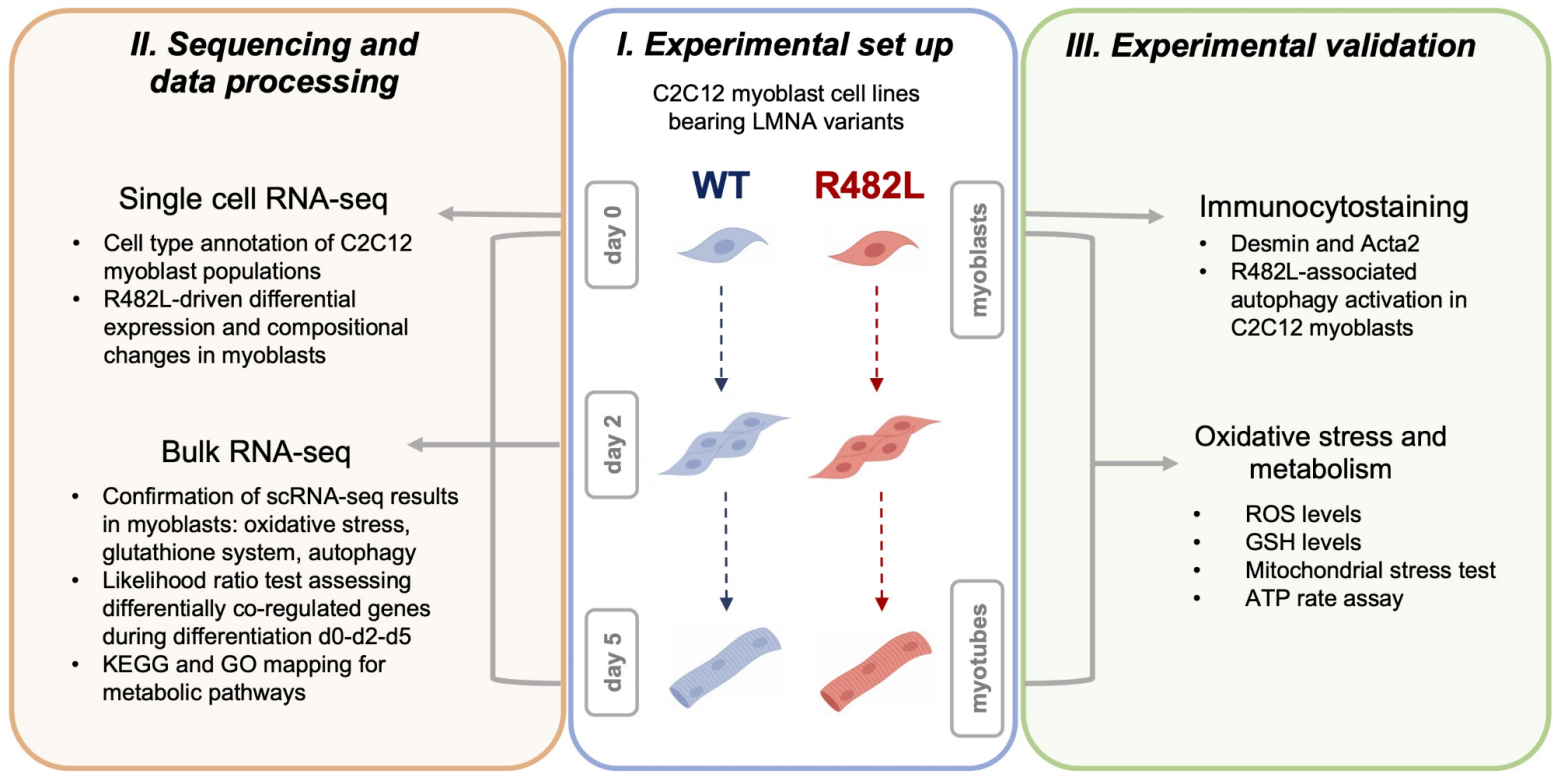
Overall design of the project. (I) C2C12 murine myoblast cell lines with human LMNA variant of wild-type (WT) or bearing R482L mutation were differentiated for 5 days to form myotubes. (II) C2C12 myoblasts WT/R482L were collected for single-cell RNA sequencing; cells on days 0,2,5 were collected for bulk RNA-sequencing for the identification of R482L-induced alterations in gene expression. (III) Validation experiments were performed for WT/R482L C2C12 myoblasts and myotubes.

In the present project, we employ a combination of single-cell transcriptome analysis, bulk RNA-seq analysis, and the measurement of cellular metabolic parameters and ROS to reveal the connections between metabolic abnormalities associated with hLMNA-R482L mutation and functional and expressional alterations in a population of skeletal muscle myoblasts. Additionally, we provide the evidence and detailed description of the heterogeneity of the C2C12 myoblast cell line, specialized independently of external stimuli. The design of the project is shown in Fig. 1.

## Results

### Single-cell RNA sequencing of C2C12 myoblasts bearing hLMNA-WT/R482L variants

Our previous work has shown alterations in coordination of cell cycle dynamics and muscle differentiation, mitochondrial uncoupling, and glycolytic activity in differentiated myotubes associated with LMNA-R482L mutation^18,20^. To characterize the differences on single cell level, we have performed single-cell RNA sequencing of transgenic hLMNA-WT and hLMNA-R482L myoblasts C2C12 and identified the specific cell subpopulations responsible for functional and metabolic alterations.

#### The general analysis

**Fig. 2:**
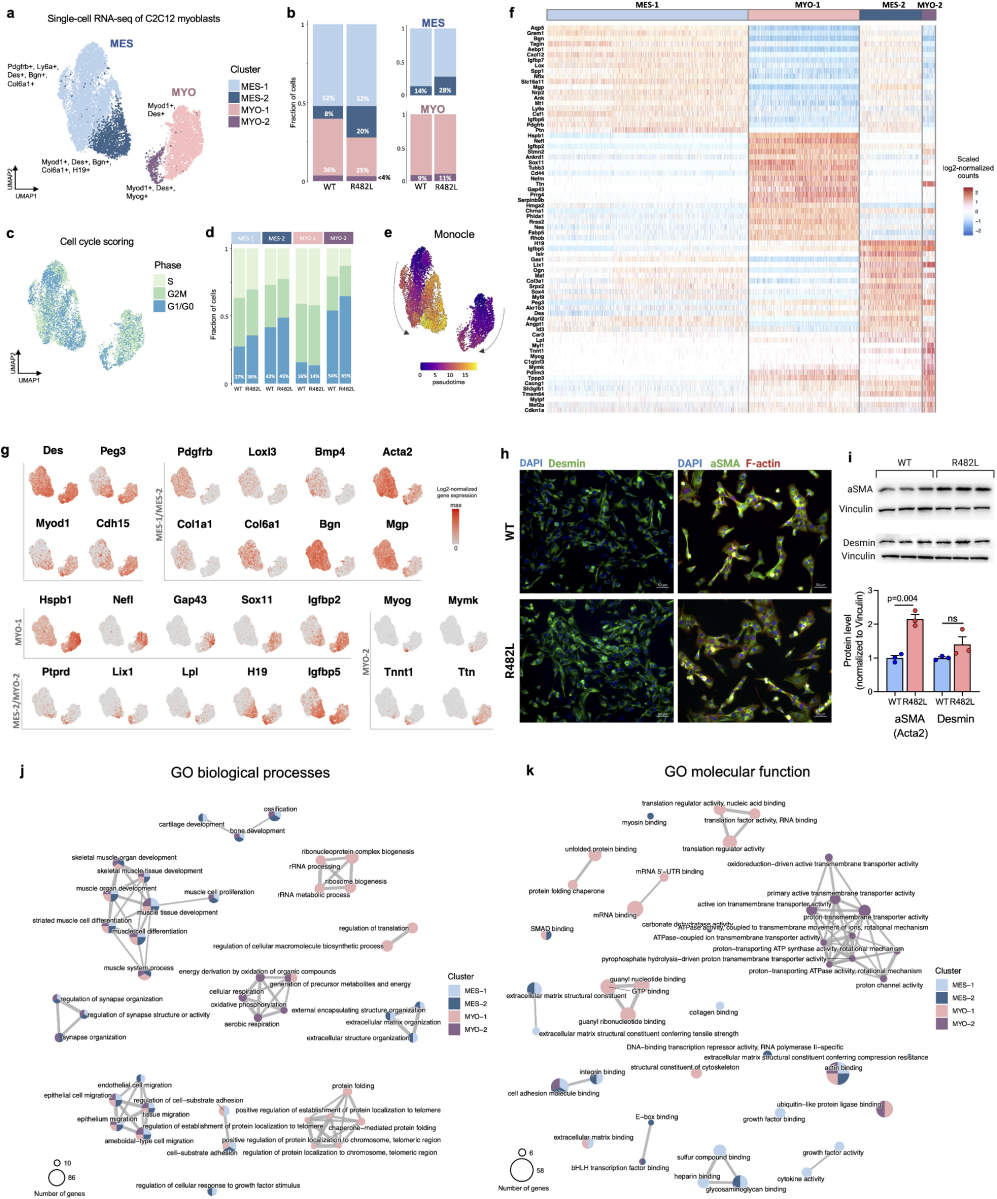
scRNA-seq of myoblasts C2C12 bearing hLMNA gene WT or R482L. (**a**) UMAP of 9470 hLMNA-WT/R482L C2C12 cells divided into 4 clusters and 2 populations MES and MYO. (**b**) Proportion of cells in clusters for WT and R482L myoblasts. (**c**) UMAP of cell cycle phase scoring. (**d**) Distribution of cell cycle phases in clusters for hLMNA-WT/R482L C2C12 myoblasts. (**e**) UMAP of cells’ pseudotime assessed with Monocle3. (**f**) Heatmap of the top 20 differentially expressed (DE) marker genes for each cell cluster in hLMNA-WT/R482L C2C12 myoblasts with log2FoldChange > 0.25, FDR = 0.01. (**g**) UMAP feature plots of the expression for some DE marker genes. (**h**) Immunocytological staining of WT and R482L myoblasts confirmed that all cells co-express Desmin and aSMA (Acta2). Scale bars represent 50 μm. (**i**) Western blotting (upper panel) analysis showed the upregulation of aSMA in R482L samples; fold changes (lower panel) of the indicated proteins relative to the WT protein expression were quantified by densitometric scanning; n = 3; ns—nonsignificant. Mann Whitney test was used. Uncropped blots images are in Fig. S2. (**j**,**k**) Pathways enriched in clusters based on DE marker genes and analyzed with Gene Ontology (GO) Biological Processes (**h**) and GO Molecular Functions (**i**) databases (FDR = 0.01).

We performed single-cell RNA sequencing of 1 sample of hLMNA-WT and 2 samples of hLMNA-R482L C2C12 myoblasts. The results of the general analysis of scRNA-Seq are summarized in Fig. 2. In total 9470 cells (3149 for hLMNA-WT and 6321 for hLMNA-R482L) with median UMI counts per cell of 15,087 and median genes per cell of 4262 passed the filter and have been integrated for further analysis. Unsupervised clustering revealed 4 subpopulations in transgenic C2C12 myoblasts (Fig. 2a) separated into two major proliferative populations: MES, or “mesenchymal” (clusters MES-1 and MES-2) and MYO, or “myogenic” (clusters MYO-1 and MYO-2). Cluster composition analysis showed that hLMNA-R482L myoblasts are enriched for cluster MES-2 (20% vs 8% in WT among all cells) but no difference in MYO composition was found (Fig. 2b, Fig. S1).

All clusters of C2C12 myoblasts contain cells being in different phases of cell cycle (Fig. 2c) with the biggest fraction of G1/G0 cells in the clusters MES-2 (48%) and MYO-2 (61%) (Fig. 2d). hLMNA-R482L samples contain more cells in G1/G0 cell cycle phase than hLMNA-WT in 3 out of 4 clusters (Fig. 2d). Single-cell trajectory analysis using Monocle suggested that cell clusters MES-2 and MYO-2 are composed of more differentiated cells (Fig. 2e), which we describe in more detail below.

#### Cluster-specific markers and associated signaling pathways of C2C12 myoblasts

Both wildtype and R482L cells exhibited skeletal muscle/striated muscle tissue signatures (Des, Myod1, M-Cadherin/Cdh15), indicating that all clusters are composed of cells of myogenic nature. The specific markers identified for each subpopulation (Fig. 2f,g, Supplementary Table S1) were, in general, in good agreement with previously published data that describe the heterogeneity of skeletal muscle precursor cells using scRNA-Seq/snRNA-Seq in vitro and in vivo (reviewed in^21^).

Cells in both MES clusters (MES-1 and MES-2) express along with myogenic marker Des and smooth muscle actin Acta2 signatures of extracellular matrix (ECM) proteins (Ccn1, Igfbp7, Loxl3) and collagens (Col1a1/2, Col3a1, Col5a2, Col6a1/2/3, Col8a1, Col12a1) indicating the dual myogenic and interstitial muscle fibroblasts (IMFs) identities of MES cells^26,27^. Additionally, in both MES clusters we detected signatures of non-myogenic mesodermal cells: Prrx2, a marker of embryo mesenchyme, a positive regulator of mesenchymal cell proliferation; markers of fibro-adipogenic precursors (FAPs) Ly6a and Pdgfrb; proteoglycans and proteoglycan’s regulators, involved in the control of bones, muscle and cartilage growth and development (Bgn, Ogn, Adamts5, Spp1); bone morphogenetic proteins (BMPs) and BMP antagonists (Bmp4, Grem1, Mgp, Crim1). Based on the cluster-specific expression profiles we have identified MES-1 as mesenchymal, and MES-2 as myofibroblast cells.

The most informative difference between MES-1 and MES-2 clusters appears to be related to the expression of myogenic markers Myod1, Cdh15 and Peg3 in the cluster of MES-2 myofibroblast: the activation of Myod1 should suppress the connective tissue potential and seal the myogenic fate along with Cdh15, while the expression of Peg3 regulates skeletal muscle lineage commitment and is expressed in both muscle satellite cells and interstitial progenitor cells of non-muscle nature^28^. Indeed, cluster MES-2 shares 17% of marker genes with MES-1, and 37% of genes with the cluster of mature myocytes (MYO-2, described below), providing additional support to the idea that there are cells with dual identities in both MES-2. Together, the observations described above indicate that the MES subpopulation consists of the cells that express markers of mesenchymal lineages, bone, muscle, cartilage, and IMFs.

Clusters in MYO were identified as cycling myoblasts (MYO-1) and mature myocytes (MYO-2). Cells in these clusters possess distinct signatures connected to the general aspects of skeletal muscle growth and development, as well as of the maturation of nuclei with specific functions in NMJ and MTJ.

In the cluster of cycling myoblasts (MYO-1, which constitutes most of the MYO cluster) among top differentially upregulated cluster-specific genes there are genes associated with various features of skeletal muscle cell biogenesis and structure. These include: (i) the actin filament cross-linking (Flnc, Flnb); (ii) maintenance and formation of NMJ (Hspb1, Nefl, Nefm, Gap43, Stmn2, Sox11, Nes) including acetylcholine receptor (AChR) subunit genes (Chrna1, Chrnb1, Chrnd, Etv4); (iii) the formation of MTJ (Ankrd1, Tnc, Itga7, Itgb1); (iv) satellite-cell-mediated skeletal muscle regeneration (Hmga2, Igfbp2, Ifrd1).

The cells from the cluster of MYO-2 exhibit the expression signatures of more advanced stages of myogenesis (Myog, Mymk, Tnnt1, Tnnt2, Ttn, Trdn) along with some MTJ markers (Lama2, Itga7) and NMJ markers (Chrna1, Chrnd, Chrng, Musk, Lrp4). Additionally, this cluster shares with the cluster MES-2 several genes related to the skeletal muscle development and structure: cells in both clusters express markers of NMJ/synapse organization (Ptprd, Tanc1/2), genes that regulate myoblasts fusion and myotube formation in differentiating myoblasts (Igfbp5, Lix1)^29,30^, lipid metabolism (Lpl)^31^ (Fig. 2g). Both MYO-2 and MES-2 share the expression of H19, the long non-coding RNA, known to play an important role in cellular differentiation: H19 is abundant in embryonic tissues, but shortly after birth is significantly downregulated in all tissues but not skeletal muscle, where it is involved in the promotion of myoblasts differentiation and muscle regeneration mediated by the microRNAs embedded within it^32^. Furthermore, it was recently shown that H19 may function as a switch between osteoblast and adipocyte differentiation in bone marrow mesenchymal stem cells (BMSC), with H19 overexpression inhibiting the differentiation of adipocytes^33,34^.

The immunostaining for Desmin confirmed the skeletal muscle nature of all cells in both WT and R482L C2C12 myoblasts (Fig. 2h). Bearing the fact that scRNA-seq compositional analysis showed that MES population is enriched with MES-2 myofibroblasts in R482L samples (Fig. 2b) we performed immunostaining and western blot analysis for aSMA (Acta2) marker of MES population (Fig. 2h,i, Fig. S2). The expression of aSMA protein is substantially higher in the R482L C2C12 myoblasts (Fig. 2j) confirming the overrepresentation of MES population in R482L cells.

The regulatory pathways associated with marker genes enriched in clusters were analyzed with Gene Ontology (GO) Biological Processes and GO Molecular Functions databases (Fig. 2j,k). Two groups of pathways were detected in all four clusters: the pathways related to skeletal muscle development and differentiation, and the pathways that regulate cell migration, including ameboidal-type migration common for myoblasts activated to fuse with pseudopodial sprouts.

We also detected both MES/MYO-specific and cluster-specific regulatory pathways (Fig. 2j,k). Thus, pathways exclusively associated with subpopulation MES regulate ECM matrix structure and organization, and cartilage development. No distinct cluster-specific regulatory pathways were detected in MES. On the contrary, in MYO population-specific regulatory pathways were detected in both clusters. In the cluster of mature myocytes, there is a group of genes that control cellular and aerobic respiration, energy production, and mitochondrial electron transport. Importantly, genes that regulate the pathways related to the control of cellular energy production were co-expressed with genes associated with more advanced steps of myogenesis. The metabolic switch in this cluster may be related to the metabolic reprogramming required for the transition from myoblasts to myotubes. The pathways specific to the cluster of MYO-1 were related to protein synthesis, protein folding, and quality control, ribosome biogenesis, all important in the regulation of skeletal muscle mass increase and maintenance.

#### hLMNA-WT/R482L myoblast cell-type specific gene expression

**Fig. 3:**
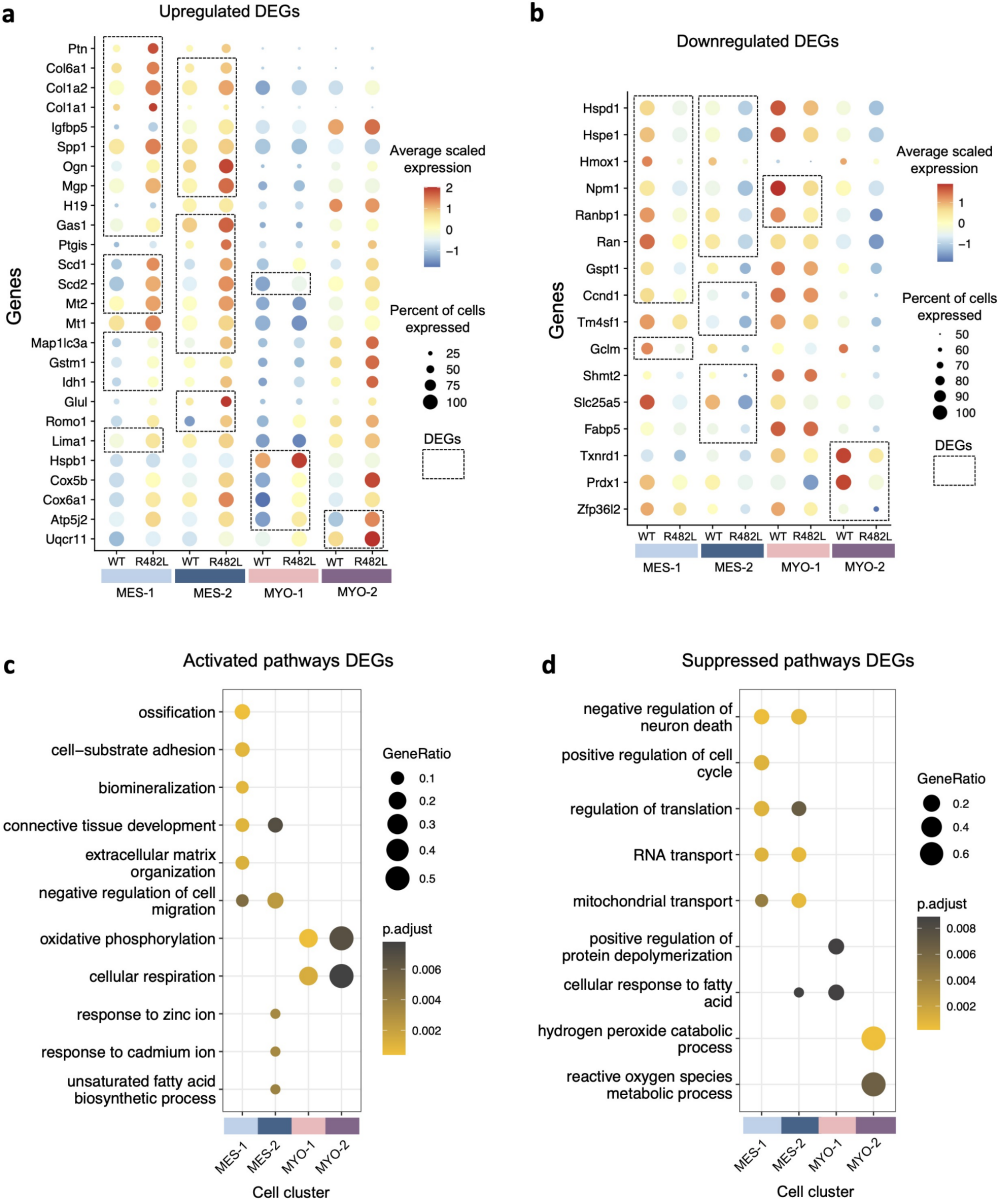
Differential gene expression at the single cell level between hLMNA-R482L and hLMNA-WT C2C12 myoblasts. (**a**,**b**) Dot plot of highlighted DEGs upregulated (**a**) and downregulated (**b**) in LMNA-R482L C2C12 cells per cluster (FDR = 0.01, |log2FoldChange|> 0.25); dashed rectangles show DEGs found within each cluster. (**c**,**d**) Corresponding highlighted activated and suppressed pathways from GO biological processes database (FDR = 0.01).

To find hLMNA-R482L-induced alterations in gene signatures we performed differential expression analysis between hLMNA-WT and hLMNA-R482L myoblasts in each cluster followed by analysis of pathways associated with differentially expressed genes (DEGs) enriched in clusters. Highlighted DEGs and corresponding pathways are summarized on Fig. 3, with the full tables of DEGs and pathways in Supplementary Table S2.

The vast majority of the expression changes were detected in the MES. In both MES-1 and MES-2 most of the upregulated in hLMNA-R482L DEGs, such as Mgp, Ogn, Spp1, Igfbp5, Col1a1, Col6a1, Ptn, are related to connective tissue development, ossification, biomineralization, negative regulation of cell migration and locomotion (Fig. 3a,c). Accordingly, in MES several DEGs and corresponding pathway related to the cell cycle progression were found downregulated in hLMNA-R482L cells: Ccnd1, Gspt1, Ran, Ranbp1 (Fig. 3b,d), while the expression of Gas1 (growth arrest specific 1) gene was upregulated. Among pathways downregulated in both MES-1/MES-2 clusters, there are mitochondrial transport, negative regulation of neuron apoptotic process, and RNA transport (Fig. 3d). In the MES-2 fatty acid biosynthesis and response to cadmium and zinc ions pathways were upregulated (Fig. 3c).

In the population MYO the MYO-2-specific downregulated pathways were associated with oxidative stress defense systems (reactive oxygen species metabolic process, hydrogen peroxide catabolic process), while upregulated pathways myocyte cluster shared with MYO-1 (oxidative phosphorylation, cellular respiration). The pathways specifically downregulated in MYO-1 were associated with response to fatty acid and protein depolymerization. None of the C2C12 clusters showed differential expression of muscle-specific transcription factors or myofiber structure genes.

Analyzing the alterations in signaling pathways associated with LMNA-R482L mutation we have noticed that many of the mechanisms behind the detected changes were related to different aspects of cellular metabolism. Since the biological features of FPLD2 are related to metabolic alterations including insulin resistance, mitochondrial dysfunction and a reactive oxygen species (ROS) increase in the cytoplasm^11^ we have examined the “metabolic and stress connections” of the specific DEGs associated with each C2C12 cluster. The possible links with skeletal muscle complications in FPLD2 patients were also considered.

In MES, four metabolism-related DEGs were upregulated (Scd1/2, Mt2, Map1lc3a) and five downregulated (Hspd1, Hspe1, Npm1, Hmox1, Ranbp1) in both clusters (Fig. 3a,b). In the MES-1 DEGs Gstm1, Idh1, and Lima1 were exclusively upregulated, and Gclm—downregulated. In MES-2 among exclusively upregulated genes were Glul, Mt1, Ptgis, and Romo1; among downregulated—Shmt2, Slc25a5, Fabp5, Tm4sf1. All listed genes are related to energy production, stress-response, stress-defense, and myopathy pathogenesis.

Thus, Scd1/2 (stearoyl CoA desaturase-1/2), and Ptgis (prostaglandin I2 synthase) located in the endoplasmic reticulum, belong to the unsaturated fatty acid biosynthetic process pathway catalyzing reactions involved in the synthesis of cholesterol, steroids, and other lipids^35^. Mt1/Mt2 (metallothioneins 1/2) are involved in defense against metal ions and oxidative stress^36^. Map1lc3a (autophagy-related ubiquitin-like modifier LC3 A) is involved in the formation of autophagosomes, and among Map1lc3a-related pathways are selective autophagy and mitophagy, alterations in both are known as important contributors to myopathy pathogenesis^37^. The mitochondrial heat shock proteins belonging to the chaperonin family, Hspd1 (Hsp60) and Hspe1 (Hsp10), both promote the proper assembly of unfolded polypeptides generated under stress conditions in the mitochondrial matrix^38^. Npm1 (nucleophosmin 1), Hmox1 (HO-1) and Ranbp1 are oxidative stress responsive genes^39–41^.

Gstm1 (glutathione S-transferase), Idh1 (isocitrate dehydrogenase 1 (NADP +)), Gclm (glutamate-cysteine ligase modifier subunit), and Glul (Glutamate-Ammonia Ligase/Glutamine Synthase) are the members of glutathione metabolic process that regulate response to increased ROS levels. Romo1 (reactive oxygen species modulator 1)^42^, Shmt2, and Slc25a5 are mitochondrial proteins that participate in oxidative phosphorylation. Lima1 inhibits actin filament depolymerization and plays a role in cholesterol homeostasis^43^. Fabp5 participates in fatty acid (FA) transport into FA-consuming tissue including skeletal muscle and heart^44^. The reduction of Tm4sf1 is involved in cell cycle arrest and apoptosis possibly via the upregulation of reactive oxygen species (ROS)^45^.

In the MYO among upregulated DEGs detected in MYO-1, two were related to mechanisms associated with cellular/endoplasmic reticulum stress: Scd2, described above, and Hspb1 (Heat Shock Protein Family B (Small) Member 1) involved in AMPK/HSPB1-mediated inhibition of inflammation and ER stress in skeletal muscle under hyperlipidemic conditions^46^.

There were only four downregulated genes in the MYO-2, and three of those DEGs are closely related to the cellular bioenergetics and stress defense: Prdx1 (peroxiredoxin-1) and Txnrd1 (thioredoxin reductase 1) are the members of antioxidant system and have reductase activity on ROS^47,48^. Zfp36l2 alters mitochondrial fission and fusion and is involved in the mechanisms determining the fate of Pax7-expressing cells during the myogenic process^49^.

Summarizing this part, hLMNA-R482L C2C12 myoblasts compared with WT show the expressional shift toward differentiation or specialization, suspending cell division and increasing the expression of connective tissue genes predominantly in the MES. Furthermore, we revealed the upregulation of genes and pathways related to the unsaturated fatty acid biosynthesis and oxidative stress defense mechanisms via glutathione and metallothioneins. Meanwhile, we detected the downregulation of mitochondrial transport, chaperonins, and expression of some genes known to be protective against ROS.

### Cellular redox homeostasis and autophagy are dysregulated in hLMNA-R482L C2C12 cells

To validate the results of scRNA-Seq data we re-analyzed our bulk RNA-seq dataset GSE150365 of hLMNA-C2C12 differentiating cells^18^ with a focus on the regulation of metabolism, bioenergetics, and stress defense in hLMNA-R482L/WT myoblasts.

**Fig. 4:**
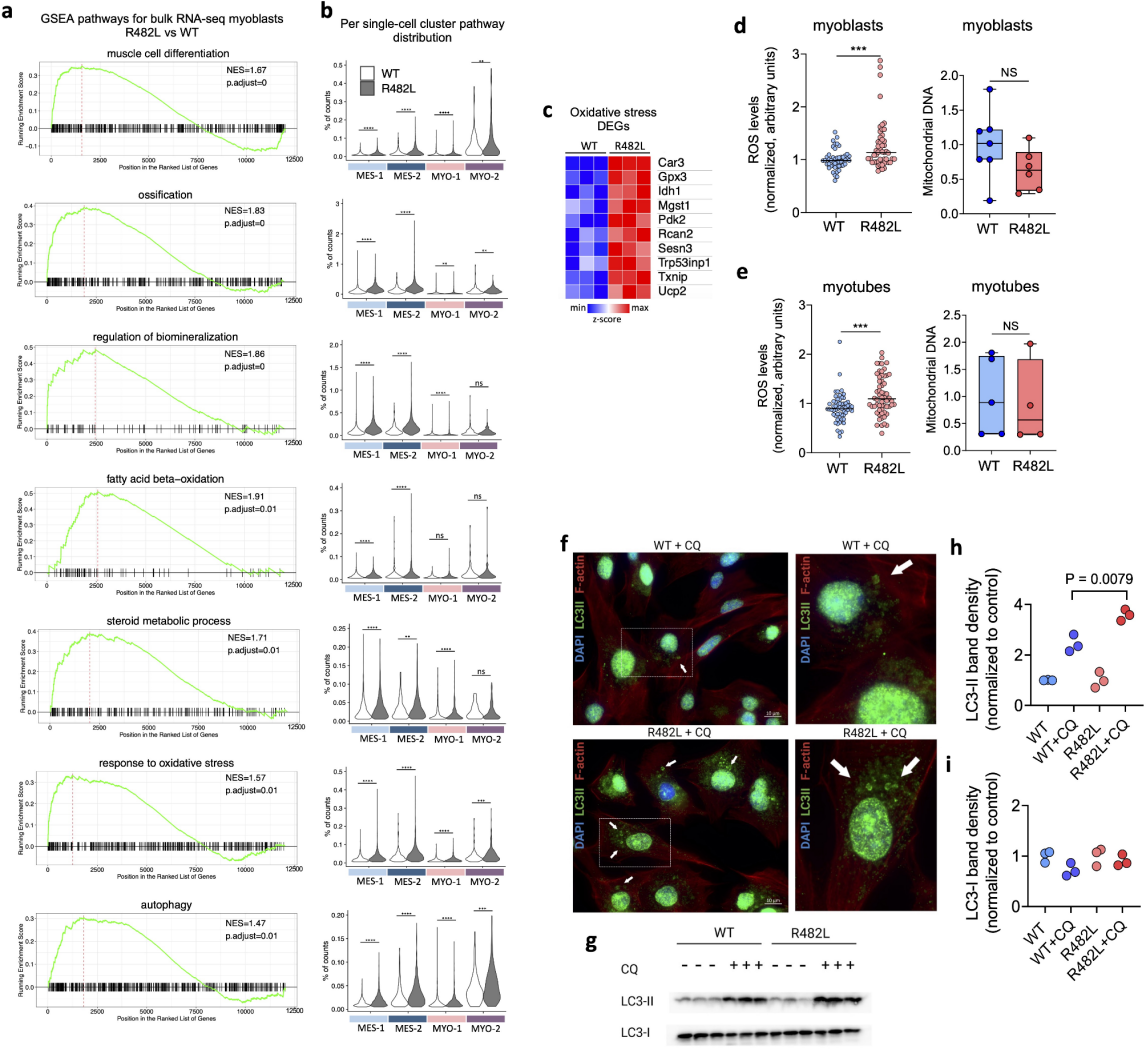
Cellular response to oxidative stress and autophagy are activated in C2C12 cells expressing the hLMNA-R482L pathological variant. (**a**) GSEA enrichment plots of the Gene Ontology pathways over ranked genes between hLMNA-R482L and hLMNA-WT (p-adjusted values and normalized enrichment scores (NES) are shown). (**b**) Distribution of pathways from (**a**) among C2C12 myoblasts’ single-cell clusters using percentage of counts of bulk RNA-seq DEGs from these pathways (**p < 0.01, ***p < 0.001, ****p < 0.0001, ns—nonsignificant); Wilcoxon rank sum test was used. (**c**) Heatmap of normalized scaled RNA-seq counts of the upregulated DEGs in hLMNA-R482L C2C12 myoblasts found in response to oxidative stress pathway from (**b**). (**d**,**e**) ROS levels in myoblasts (**d**) and myotubes (**e**) using fluorescein (DCF), an indicator of ROS in living cells (n > 30, **p < 0.01, ***p < 0.001); mitochondrial DNA measured using qPCR (n > 4, NS—nonsignificant); Mann–Whitney statistical test was used. (**f**) Immunocytological staining of WT and R482L myoblasts treated with autophagic flux inhibitor chloroquine (CQ) shows LC3 staining enriched with LC3-II (green) isoform; F-actin (red); DAPI (blue). White arrows indicate the accumulation autophagic-related structures. (**g**) Western blotting for Cytosolic (LC3-I) and membrane-associated (LC3-II) isoform in R482L/WT myoblasts was used to estimate the abundance of autophagic-related structures before the degradation. Ponceau S staining served as a loading control. Uncropped blot images are in Fig. S2. (**h**,**i**) Fold changes of LC3-II (**h**) and LC3-I (**i**) proteins relative to the control band (non-treated with CQ) were determined by densitometric scanning.

In total 192 upregulated and 31 downregulated DEGs with |Fold Change|> 1.5 and False Discovery Rate (FDR) as 0.05 were found in hLMNA-R482L myoblasts (Fig. S3, Supplementary Table S3). As was shown previously, Gene Set Enrichment Analysis (GSEA) revealed the upregulation of GO pathways related to myogenesis (Fig. 4a) and downregulation of cell cycle pathways (Supplementary Table S3) in hLMNA-R482L. In addition, we found the upregulation of ossification, regulation of biomineralization, fatty acid beta-oxidation, steroid metabolic process, autophagy, and response to oxidative stress pathways in hLMNA-R482L myoblasts with FDR < 0.05 (Fig. 4a). To visualize the distribution of pathways among single-cell clusters we calculated the percentage of counts for bulk RNA-seq DEGs from these pathways for each cell (Fig. 4b). This showed that: (i) as expected, the mutation-induced upregulation of muscle cell differentiation was detected in MYO-2 and MES-2, the latter one cluster—overrepresented in hLMNA-R482L sample; (ii) almost no differences were detected between hLMNA-R482L/WT myoblasts in MYO-1 (cycling myoblasts); (iii) the upregulation in hLMNA-R482L samples pathways related to connective tissue (ossification, biomineralization) and steroid metabolic process was associated with MES subpopulation; (iv) hLMNA-R482L-induced upregulation of the response to oxidative stress and autophagy was mostly associated with differentiating clusters MES-2 and MYO-2 and to some extent with MES-1. Lipid- and stress-related pathways represent not crossing processes as shown on a network plot for GSEA core enriched genes (Fig. S3). Interestingly, we did not find in scRNA-seq the differential expression of myogenic-specific genes such as Myog, Mymk, myosin heavy and light chains, in contrast to bulk RNA-seq. Thus, the upregulation of myogenesis-related processes in some C2C12 clusters (Fig. 4b) could be explained by the increased expression of the others myogenesis-related genes that play a supportive role in muscle fiber formation and growth.

The specific DEGs associated with oxidative stress pathway are shown on Fig. 4c. Most of these genes are known to control ROS accumulation and/or scavenging through different molecular mechanisms. Thus, Trp53inp1 in early myogenesis regulates the balance between apoptosis and cell cycle exit^50^, and is involved in control of intracellular ROS levels under normal conditions or after oxidant challenge^51^. Txnip overexpression in skeletal muscle negatively regulates insulin sensitivity and glucose uptake^52^, and inhibits the activity of antioxidant defense Trx-1 system^53^. Gpx3, the only secreted glutathione peroxidase, scavenges H_2_O_2_ to reduce systemic oxidative stress in human skeletal muscle and maintains cell homeostasis in stress condition^54^. Stress-response gene microsomal glutathione S-transferase (Mgst1) is well known for glutathione transferase and glutathione peroxidase activities^55^, and shown to be activated in vitro by ROS and reactive nitrogen species^56^. Sesn3 belongs to a family of stress-sensitive genes, regulates lipid metabolism^57^, and plays a major role in reestablishing the antioxidant thioredoxin/thioredoxin reductase defense system^58^. Idh1 catalyzes the oxidative decarboxylation of isocitrate to α-ketoglutarate and NADPH which is an essential cofactor for the biosynthesis of GSH which is the most abundant cellular antioxidant. The uncoupling protein 2 (Ucp2) is highly expressed in the heart and skeletal muscle where its expression is activated by free fatty acids. It is involved in the control of cellular metabolism and defense against ROS in glycolytic cells acting through the inhibition of pyruvate transport into mitochondria, which is compensated by fatty acids oxidation^59^.

Then, we measured ROS levels in the course of myogenesis and detected its increased accumulation in both hLMNA-R482L myoblasts and differentiated myotubes (Fig. 4d,e). The mitochondrial content did not differ between hLMNA-WT and hLMNA-R482L samples suggesting that the changes in ROS were connected to metabolic alterations, not to the differences in mitochondrial content (Fig. 4d,e). It is noteworthy, that we have shown above (Figs. 3a,b, 4b) that stress-response genes were dysregulated in MES-1/2 and MYO-2 clusters in hLMNA-R482L myoblasts.

One more set of important data came from this experiment: the analysis of bulk RNA-Seq showed the upregulation of autophagy pathway in hLMNA-R482L myoblasts (Fig. 4a), and the distribution of pathway among single-cell clusters also showed the mutation-related upregulation (Fig. 4b). Since it is known that autophagy is a dynamic process that controls the balance of cellular energy metabolism and contributes to myopathy pathogenesis^60^, we tested if autophagy was activated in LMNA-R482L myoblasts in vitro (Fig. 4f–i). Indeed, both immunofluorescence staining and western blotting (WB) analysis of LC3-II, a hallmark of autophagosome, confirmed autophagy activation in LMNA-R482L myoblasts: in the presence of autophagic flux inhibitor chloroquine (CQ) the accumulation of LC3-positive structures was observed (Fig. 4f), and the accumulation of the LC3-II isoform was significantly higher in LMNA-R482L myoblasts as detected by WB analysis (Figs. 4g–i, S2).

### Glutathione network alterations in C2C12 myoblasts bearing pathological hLMNA-R482L

**Fig. 5:**
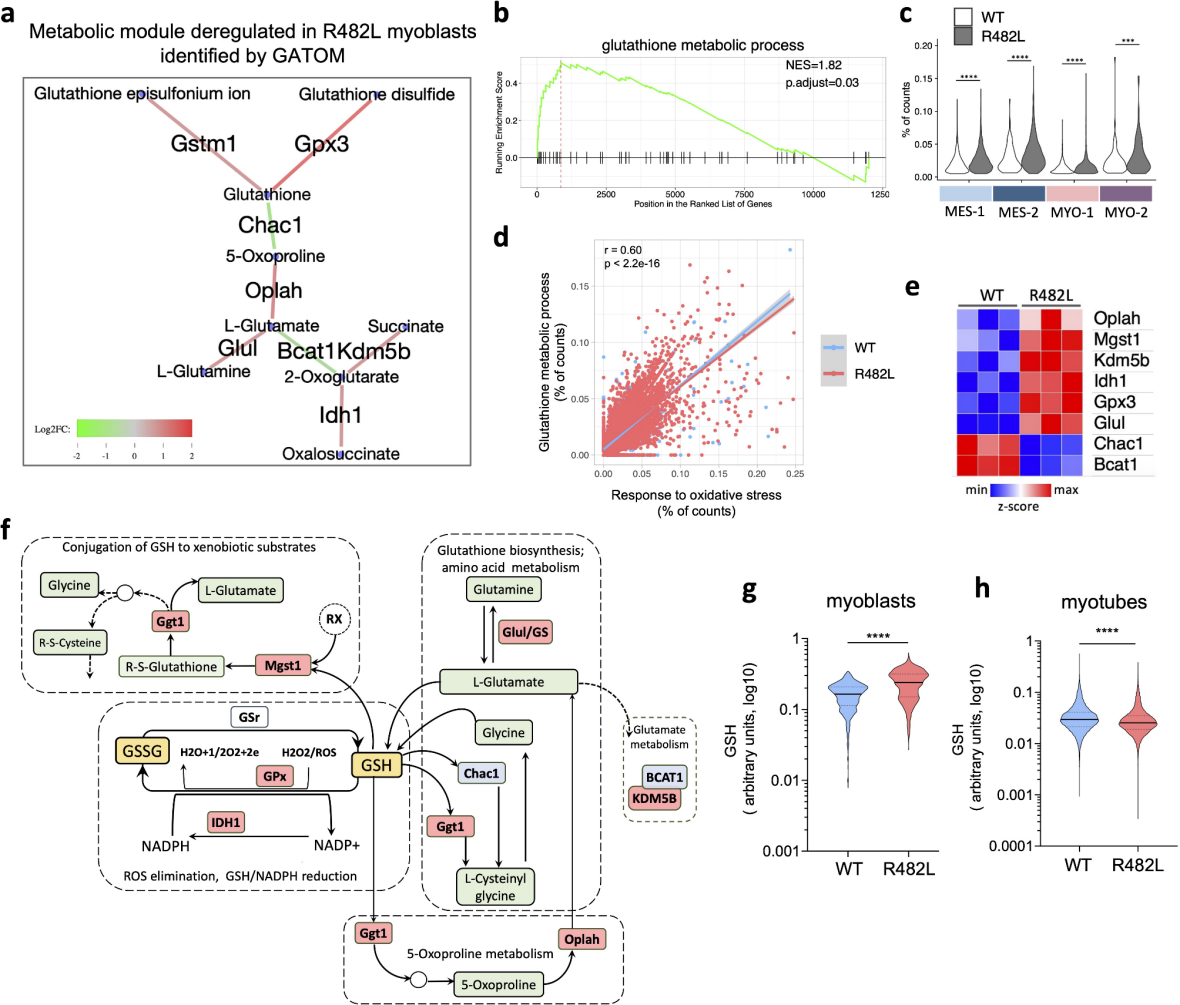
Alterations in the glutathione network associated with the hLMNA-R482L myoblasts result in increased GSH levels. (**a**) The result of GATOM web-application on DEGs in hLMNA-R482L/WT myoblasts revealed the metabolic submodule related to glutathione-mediated (GSH/GSSG) cellular redox network in R482L. (**b**) GSEA enrichment plot of the GO glutathione metabolic process pathway over ranked genes between hLMNA-R482L and hLMNA-WT myoblasts. (**c**) Distribution of pathway from (b) among C2C12 myoblasts’ single-cell clusters using percentage of counts for upregulated DEGs from (e); (**p < 0.01, ***p < 0.001, ****p < 0.0001, ns—nonsignificant); Wilcoxon rank sum test was used. (**d**) Pearson’s correlation for C2C12 myoblasts’ single cells between the percentages of counts for response to oxitative stress and glutathione metabolic process pathways. Parameters are shown for all cells since no difference between WT and R482L conditions was found. (**e**) Genes visualized as a heatmap of normalized scaled counts that displays up/downregulation in C2C12 myoblasts expressing hLMNA-R482L (FDR = 0.05). (**f**) Simplified scheme of the alterations in KEGG glutathione metabolism pathway (map00480) associated with hLMNA-R482L variant. GSH/GSSG—reduced/oxidized forms of glutathione; the enzymes which expression is upregulated in hLMNA-R482L myoblasts are highlighted in red, downregulated—in blue; GPx—glutathione peroxidase; IDH1—isocitrate dehydrogenase (NADP( +)); GSr—glutathione-disulfide reductase; Mgst1—microsomal glutathione S-transferase 1; Oplah—5-oxoprolinase, ATP-hydrolyzing; gamma-glutamyl-transferase 1; Chac1—glutathione specific gamma-glutamyl-cyclo-transferase 1; Glul—glutamate-ammonia ligase; Bcat1—branched chain amino acid transaminase; Kdm5b—lysine demethylase 5B. (**g**,**h**) GSH levels in the living C2C12 myoblasts (g) and myotubes (h) expressing hLMNA-WT and hLMNA-R482L calculated as F510/F580 ratio (n > 4000, ****p < 0.0001).

Among antioxidant defense systems, the glutathione (reduced/oxidized GSH/GSSG) and thioredoxin/thioredoxin reductase (Trx/TrxR) systems are the most important. Using GATOM web-application (GATOM: integrated analysis of genes and metabolites)^61^ and DEGs from bulk transcriptome sequencing data of myoblasts, we have shown that most regulated and unique metabolic submodule was directly related to GSH/GSSG cellular redox network in hLMNA-R482L (Fig. 5a), and GSEA analysis revealed upregulation of the GO glutathione metabolic process pathway (Fig. 5b). The distribution of related bulk DEGs among single-cell clusters showed that the upregulation of pathway was associated with MES-1/2 and MYO-2 (Fig. 5c). The correlation for the percentage of counts between glutathione metabolic process and response to oxidative stress pathways was also found (r = 0.60, p < 2e−16) (Fig. 5d).

Up- and down- regulated DEGs present in both metabolic submodule and glutathione pathway visualized as a heat map on the Fig. 5e. As detailed above, Gpx3, Idh1, and Mgst1 genes act as a part of the glutathione antioxidant defense system. Chac1 is a glutathione degrading enzyme that regulates the GSH/GSSG ratio in the living cells^62^; the glutamine synthetase GS/Glul negatively regulates GSH production due activation of synthesizes of glutamine (Gln) from glutamate (Glu) which may result in downregulation of GSH production^63^; Oplah (5-oxoprolinase) is an enzyme involved in the γ-glutamyl cycle, and functions by converting 5-oxoproline, a degradation product of glutathione, to glutamate for de novo GSH synthesis^64^.

The role of branched-chain amino acid aminotransferase 1 (Bcat1) in regulation of redox homeostasis is also well known: Bcat1-engaged branched-chain amino acid metabolism involves two diverse pathways: one of those generates GSH by the glutamate-cysteine ligase catalytic subunit (GCLC), the rate-limiting enzyme of GSH synthesis, and the other produces coenzyme A compounds by branched-chain keto acid dehydrogenase complex (BCKDK), which participates in the tricarboxylic acid (TCA) cycle^65^. Finally, there are data that ROS might modulate the histone methylation balance to modify the chromatin accessibility in response to stimuli, and Kdm5b (lysine demethylase 5B) is capable of repressing transcription by demethylating the activating marks H3K4me2/3 in response to ROS, NO and hypoxia. Additionally, overexpression of KDM5b was shown to be accompanied by an enhanced GSH-dependent redox capacity^66^. In total, on the Fig. 5f we visualized the alterations in the glutathione network in hLMNA-R482L C2C12 myoblasts that might possibly result in alterations in cellular GSH-related mechanisms of enzymatic detoxification of ROS.

To make sure that the glutathione network is affected in hLMNA-R482L C2C12 myoblasts we also tested in vitro GSH levels. The results were in a good agreement with data shown on glutathione network scheme: GSH levels in the living C2C12 myoblasts expressing hLMNA-R482L pathological variant were significantly higher than in WT (Fig. 5g). However, in differentiated hLMNA-R482L C2C12 myotubes GSH levels were decreased (Fig. 5h).

Thus, we show that the glutathione-mediated antioxidant defense system in hLMNA-R482L myoblasts was activated to increase the production of GSH, but the activation was not efficient to stabilize the ROS content at the levels compatible to ones in WT (Fig. 4d). Moreover, in differentiated myotubes we also detected the increased levels of ROS but decreased levels of GSH in hLMNA-R482L samples (Figs. 4e, 5h), suggesting the failure of stress-defense system in mature myotubes.

### The regulation of carbohydrate metabolism is severely affected in differentiating C2C12 myoblasts expressing the hLMNA-R482L variant

**Fig. 6:**
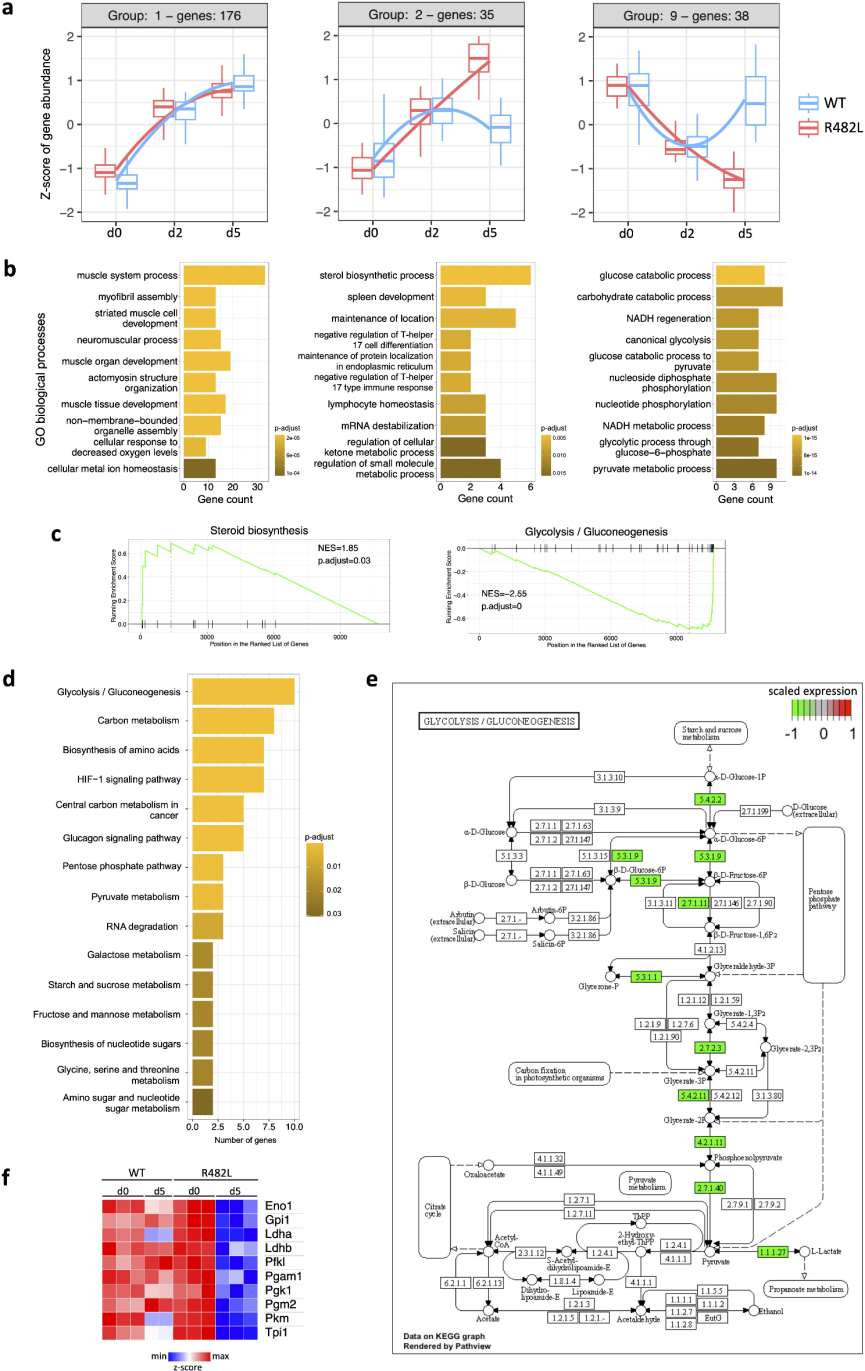
Carbohydrate metabolism is severely affected in differentiating hLMNA-R482L C2C12 myoblasts. (**a**) The results of Likelihood Ratio Test (LRT) assessing differentially co-regulated gene groups in course of myogenic differentiation (days 0, 2, 5) of hLMNA-R482L/WT C2C12 myoblasts (FDR = 0.01); all group clusters and list of genes are on Fig. S4, Table S4. (**b**) Corresponding top 10 signaling pathways from Gene Ontology (GO) biological processes database associated with genes co-localized in groups 1,2 and 9; FDR = 0.05. (**c**) GSEA enrichment plots of the KEGG pathways over ranked genes between hLMNA-R482L and hLMNA-WT myotubes. (**d**) KEGG pathways associated with genes co-localized in group 9 (FDR = 0.05). (**e**) KEGG mapping for the glycolysis/gluconeogenesis pathway; genes co-localized in group 9 are shown in green indicating its downregulation in hLMNA-R482L myotubes, and (f) visualized as a heat map of normalized scaled RNA-seq counts that displays up/downregulation during differentiation; genes’ names and functions are given in the Table 1.

Given the facts that hLMNA-R482L myoblasts accumulate ROS and activate stress-defense system we examined whether the dynamics of differentiation was affected. The bulk RNA-seq data described above^18^ was analyzed during the myoblasts-to-myotubes transition at three checkpoints: day 0 (non-differentiated myoblasts), day 2 (early step of differentiation), and day 5 (multi-nucleated myotubes). Previously we showed the imbalance in myogenesis and cell cycle exit transcriptional programs in mutant cell lines^18^. Here we decided to assess differentially co-regulated gene groups in the course of myogenic differentiation and performed Likelihood Ratio Test (LRT) followed by the clustering and analysis of signaling pathways associated with genes in each cluster (Figs. 6, and S4). We uncovered 9 groups of differentially co-regulated genes (FDR = 0.01). The most informative findings were detected in groups 1, 2, and 9 (Fig. 6a,b); the whole list of group-specific genes is given in Supplementary Table S4.

In group 1 were co-localized genes associated with pathways that regulate multiple aspects of skeletal muscle growth and development (Fig. 6a,b). The expression of genes in this cluster stably increased during differentiation in both lines of myoblasts, in good accordance with dynamics of myotubes development and our previous data^18^. The expression of genes in group 2 did not differ between myoblasts at day 0, increased in both cultures by day 2 after stimulation of myogenesis, and continued to increase up to day 5 in hLMNA-R482L, but not in hLMNA-WT (Fig. 6a). Genes in this group were associated with the sterol biosynthetic process, regulation of ketone metabolism, and mRNA destabilization (Fig. 6b).

The expression of genes from group 9 showed a strong difference between the two lines by day 5 (Fig. 6a,b). These genes were associated with the regulation of carbohydrates metabolism, and bioenergetic (ATP production, NADH regeneration, etc.), which indicate the mutation-induced alterations in adaptive metabolic reprogramming required during transition from myoblasts to myotubes^16,67,68^. Analysis of KEGG pathways using GSEA in hLMNA-R482L myotubes compared to WT confirmed the upregulation of steroid biosynthesis and downregulation of glycolysis/gluconeogenesis pathways (Fig. 6c, Supplementary Table S3).

To specify the alterations in metabolic reprogramming during the differentiation of hLMNA-R482L myoblasts we analyzed genes in group 9 using KEGG database (Fig. 6d). Ten out of 38 genes were found to be related to the glycolysis pathway (Fig. 6e). Genes associated with group 9 are shown in green, highlighting the fact that practically all steps of glycolysis were suppressed, including two rate-limiting steps regulated by phosphofructokinase-1 (Pfkl), that phosphorylates fructose 6-phosphate into fructose 1,6-bisphosphate, and pyruvate kinase (Pkm) that catalyzes final reaction of glycolysis, the transfer of a phosphoryl group from phosphoenolpyruvate to ADP, generating ATP and pyruvate. All these genes are shown as a heat map of normalized and scaled RNA-seq counts that display up/downregulated genes at different time points during differentiation (Fig. 6f); the functions of these genes are listed in Table 1.

**Table 1:** Genes from the cluster 9 of the Fig. 6a are associated with the downregulation of glycolysis during hLMNA-R482L/WT C2C12 myoblasts differentiation.

KEGG ID	Gene symbol	Gene name	Function
5.4.2.2	Pgm2	Phosphoglucomutase 2	Catalyzes the interconversion of glucose 1-phosphate and glucose-6-phosphate
5.3.1.9	Gpi1	Glucose-6-phosphate isomerase 1	In the cytoplasm interconverts glucose-6-phosphate and fructose-6-phosphate. Extracellularly functions as a neurotrophic factor that promotes survival of skeletal motor neurons and sensory neurons
2.7.1.1	Pfkl	Phosphofructokinase	Catalyzes the phosphorylation of fructose 6-phosphate to fructose-1,6-bisphosphate, the irreversible, major rate-limiting step of glycolysis
5.3.1.1	Tpi1	Triosephosphate isomerase 1	Catalyzes the isomerization of glyceraldehyde-3-phosphate
2.7.2.3	Pgk1	Phosphoglycerate kinase 1	Glycolytic enzyme; catalyzes the conversion of 1,3-diphosphoglycerate to 3-phosphoglycerate
5.4.2.11	Pgam1	2,3-bisphosphoglycerate mutase	glycolytic enzyme; catalyzes the conversion of 3-phosphoglycerate to 2-phosphoglycerate
4.2.11	Eno1	enolase 1	Glycolytic enzyme catalyzing conversion of 2-prhosphoglycerate to phosphoenolpyruvate
2.7.1.40	PK3(PKM)	Pyruvate kinase M1/2	Catalyzes the last step of glycolysis: transfer of a phosphoryl group from phosphoenolpyruvate to ADP, generating ATP and pyruvate, the rate-limiting step of glycolysis
1.1.1.27	Ldha	Lactate dehydrogenase A	Catalyzes the conversion of L-lactate and NAD to pyruvate and NADH in the final step of anaerobic glycolysis; found predominantly in muscle tissue
1.1.1.27	Ldhb	Lactate dehydrogenase B	Catalyzes the interconversion of pyruvate and lactate with concomitant interconversion of NADH and NAD + in a post-glycolysis process

The other KEGG pathways associated with genes from group 9 control carbohydrate metabolism, indicating decreased ability of hLMNA-R482L myotubes to generate energy for the synthesis of amino acids and nucleotides as well as to provide a substrate for oxidative phosphorylation (Fig. 6d).

### hLMNA-R482L mutation-induced alterations in skeletal muscle cellular bioenergetics during differentiation

**Fig. 7:**
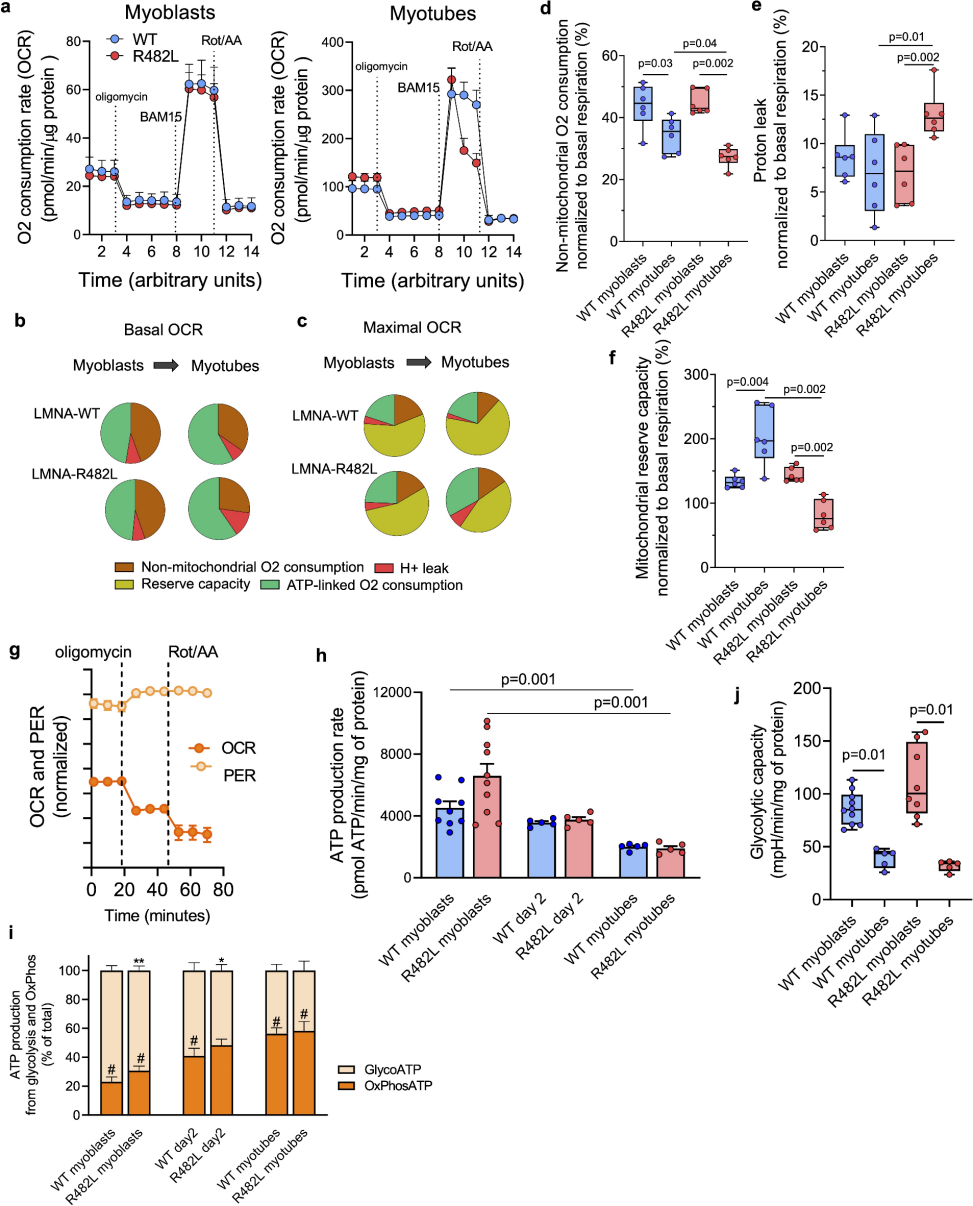
LMNA-R482L-induced alterations in cellular bioenergetics in myoblast-to-myotube transition. (**a**) Mitochondrial bioenergetic parameters were determined in myoblasts and myotubes from oxygen consumption rate (OCR) profile in the presence of mitochondrial function inhibitors. (**b**) The OCR at basal condition was established as 100%, and the proportion of ATP-linked O2 consumption, non-mitochondrial O2 consumption, and proton leak was calculated for C2C12 myoblasts and myotubes and shown as the pie charts. (**c**) The maximal OCR was established as 100% and the contribution of non-mitochondrial O2 consumption, ATP-linked OCR, proton leak, and reserve capacity were estimated. (**d**–**f**) The quantitative analysis of mitochondrial bioenergetic parameters during the transition from myoblasts to differentiated myotubes: non-mitochondrial O2 consumption (**d**), proton leak (**e**), and mitochondrial reserve capacity (**f**). Data are presented as the boxplots, min. to max., n = 6. (**g**) Real time OCR and proton efflux rate (PER) dynamics under mitochondrial function inhibitors shown for WT myoblasts sample, as an example. (**h**) Total ATP production rate estimated as a sum of GlycoATP and OxPhosATP at basal levels of PER/OCR at day 0 (myoblasts), day 2 (myoblasts fusion), and in differentiated myotubes in course of myogenic differentiation; n = 5–10. (**i**) The balance between fractions of glycolytic ATP and OxPhos ATP within total ATP production in course of differentiation; n = 5–10; GlycoATP WT vs R482L: *p = 0.03; **p = 0.005; GlycoATP vs OxPhosATP: #p = 0.0001. (**j**) The glycolytic reserve capacity calculated as the difference between PER at basal condition and PER after oligomycin was injected. Mann–Whitney statistical test was used. P-values are provided if significant.

To estimate hLMNA-R482L mutation-induced alterations in skeletal muscle cellular bioenergetics we performed Seahorse ATP Production Rate Test and Mitochondrial stress test. All parameters were estimated in both proliferating myoblasts and differentiated myotubes. The oxygen consumption rate (OCR) dynamics in the course of mitochondrial stress test is shown on Fig. 7a. While the contribution of different cellular processes to O2 consumption at basal levels did not differ significantly between lines in both myoblasts and myotubes (Fig. 7b), at maximal respiration potential sustainable by the cells, the fraction of reserve capacity increased substantially in course of differentiation in hLMNA-WT, but not in hLMNA-R482L (Fig. 7c).

The quantitation of individual parameters normalized to basal respiration showed as the box plot charts for both myoblasts and myotubes (Fig. 7d–f). Thus, during differentiation we detected the significant decrease in non-mitochondrial O2 consumption in both WT and R482L lines (Fig. 7d), while the proton leak increased significantly in hLMNA-R482L but not in hLMNA-WT myotubes (Fig. 7e). Importantly, the mitochondrial reserve capacity increased in WT myoblasts in course of differentiation, while in hLMNA-R482L myotubes we detected its significant downregulation (Fig. 7f) which usually indicates decreased substrate availability, and/or alterations in mitochondrial network and activity of individual mitochondrial complexes. These experimental data are in line with results of transcriptome analysis shown in Fig. 6, that demonstrates downregulation of pathways that control NADH regeneration and carbohydrates metabolism in differentiating samples. Additionally, the expression of genes from Slc2 (GLUT) family of membrane transporters of monosaccharides and other small carbon compounds across the membranes (Glut1/Glut3/Glut8/Glut10) was downregulated as well as genes involved in lipoprotein metabolism, cholesterol and cellular lipid homeostasis (Apoe, Abcg1, Abca1, Samd1) (Supplementary Table S3) which supports the assumption of decreased substrate availability in hLMNA-R482L myotubes.

To estimate separately the mitochondrial and glycolytic ATP production rates in C2C12 hLMNA-WT and hLMNA-R482L lines we employed Seahorse ATP Production Rate Test. Real time OCR and proton efflux rate (PER) dynamics shown for hLMNA-WT myoblasts sample as an example to illustrate the principle of calculation of the of glycolytic and OxPhos ATP fractions within total ATP production, as well as glycolytic reserve capacity (Fig. 7g). To better understand the dynamic of metabolic shift during myogenic differentiation we added an additional time point (48 h after stimulation) into analysis.

There were a lot of similarities between cell lines in ATP production profile: in course of differentiation the production of total ATP gradually decreased in both WT and R482L samples (Fig. 7h). Indeed, at first steps of myogenesis a lot of energy is needed to maintain the high proliferation rate, myoblasts fusion, and proteins synthesis; after myofiber is composed and grown, the energy demand should decrease especially in case of fiber inactivity (Fig. 7h). Also, in both WT and R482L proliferating myoblasts the fraction of glycolytic ATP was significantly larger than the fraction of OxPhos ATP within total ATP production, while OxPhos ATP fraction increased substantially in course of differentiation (Fig. 7i). However, the difference in ATP production profile between WT and R482L lines was also shown: the fraction of ATP derived from glycolysis was significantly lower in proliferating hLMNA-R482L myoblasts and during early steps of myogenesis compared with WT-LMNA myoblasts (Fig. 7i). The latter is in a good agreement with our data on hLMNA-R482L-induced downregulation of glycolysis (Fig. 6d–f), as well as with upregulated oxidative phosphorylation and cellular respiration pathways showed in MYO clusters by scRNA-Seq analysis (Fig. 3c).

Importantly, after myoblasts were stimulated to differentiate, the glycolytic capacity dropped down in both lines (Fig. 7j), but in WT samples we detected the increase in mitochondrial reserve capacity in course of differentiation, while in R482L samples the differentiation-induced metabolic switch was not shown. On contrary, the mitochondrial reserve capacity dropped down during differentiation of hLMNA-R482L myoblasts (Fig. 7f). These data are in line with the numerous studies demonstrating the reliance of proliferating myoblasts on glycolysis, with an increase in OXPHOS activity following differentiation^16^. We suggest that the alterations in differentiation-related metabolic reprogramming we have shown in vitro may be associated with the skeletal muscle abnormalities in FPLD2.

## Discussion

In this work we suggested a simple in vitro muscle model of FPLD2 and described the changes within the population of skeletal muscle C2C12 myoblasts bearing hLMNA-R482L mutation. Additionally, we provide a comprehensive description of C2C12 myoblast’s subpopulations at single cell level.

The scRNA-Seq data analysis of C2C12 myoblasts detected two major cellular subpopulations, each composed of two distinct clusters in both hLMNA-WT and hLMNA-R482L samples. These clusters showed the signatures specific for skeletal muscle progenitor cells committed to different fate within skeletal muscle fiber (Fig. 2): IMF/myofibroblasts, FAPs, pericytes, NMJ and MTJ cells^21,69,70^. Additionally, there was a small cluster of mature myocytes MYO-2 which differentiation was presumably induced spontaneously in proliferating culture due to the cell-to-cell contacts. Therefore, we have shown that myoblast fate was determined within population independently of external physiological stimuli, and cells get specified for the future functions in myofibril instructing each other through the paracrine signaling and/or contact interactions to create the population of functionally diverse cells needed for maintenances of skeletal muscle cellular complexity.

The unique gene expression patterns in myofiber nuclei were previously shown in vivo: myonuclei located at NMJ are specialized for the formation and maintenance of the synaptic apparatus^23^. These nuclei express markers similar to ones we have detected in clusters MYO-1 and MYO-2: acetylcholine receptor (AChR) subunit genes (Chrna1, Chrnb1, Chrnd, Chrng), as well as the genes having established roles in NMJ development such as Musk (postsynaptic membrane organization, patterning of skeletal muscle, anchoring of acetylcholinesterase, and guidance of motor axons), Lrp4 (regulation of presynaptic membrane organization), and Etv4 (motor neuron axon guidance) (Supplementary Table S1) (reviewed in^23^).

The muscle–tendon connection regions are also known to exhibit transcriptional specialization^21,23,26^. During embryonic development emerging cartilage immediately interacts with individual myocytes synchronizing muscle and cartilage maturation^25^. Furthermore, MTJ regions of muscles are formed by fusion of myocytes with interstitial muscle fibroblasts with dual transcriptional identity^26^. In our model myoblasts from MES subpopulation can secrete ECM components (Col1a1, Col6a3, Col3a1) into the space between cells to establish the interactions with myoblasts and/or myocytes expressing MTJ markers Itga7, Lama2, Tnc, and Ankrd1. These observations suggest that the regulatory crosstalk between cells from MES and MYO subpopulations is necessary to build the functional MTJ. Importantly, the studies by others^71^ and our previous data^18^ showed that C2C12 myoblasts constitutively express scleraxis (Scx) indicating the potential of C2C12 to transdifferentiate into tendon or, at least, tendon-like cells. Here we have shown that the pathways exclusively associated with MES regulate cartilage development (Fig. 2j,k). Of note, most of the cells that possess markers of both MTJ and NMJ are located in clusters MYO-1/2 and MES-2, which may indicate the importance of interaction of cells from these two populations in course of skeletal muscle nuclei specialization, as well as point to the possible role of dysregulations in the balanced MYO—MES cross-talk in skeletal muscle pathological alterations. Indeed, there are data on literature that the LMNA mutations–induced defects in the positioning of synaptic nuclei at the NMJ in muscle fibers contribute to the laminopathy with skeletal muscle phenotype development and progression^72^. Additionally, other publications suggested that in LMNA mutant myofibers the nuclear envelope rupture increased at MTJ compared to the muscle fiber body, which correlated with disease severity^73^.

The role of skeletal muscle interstitial cells co-expressing both mesenchymal and myogenic markers in skeletal muscle regeneration in vivo is recognized^26,70,74^. Here we call the cluster that fully fits this description “myofibroblasts” or MES-2, and this cluster is significantly overrepresented in hLMNA-R482L samples.

It is worth noticing that LMNA-R482L-related alterations were detected in all four clusters of C2C12 myoblasts, though the most dramatic changes were discovered in MES (Fig. 3). Many of these changes in both signaling pathways and cluster-specific DEGs are associated with cellular metabolism and stress response: among top DEGs up- and downregulated in hLMNA-R482 MES-2 and MYO-2 a large fraction of DEGs attenuate oxidative and/or endoplasmic reticulum stress, control protein folding-unfolding, and involved in formation of autophagosomes which is in a good agreement with the current understanding of skeletal muscle pathological phenotype in FPLD2^75^. The mechanisms involved in different aspects of cellular metabolism and stress defense regulation were considered as the targets of FPLD2 management, though no effective cure is available so far (reviewed in^76^). Our results empower the understanding how LMNA-R482L-induced changes in mesenchymal population (MES) of skeletal muscle resident cells might contribute to skeletal muscle pathological phenotype development in FPLD2.

The connections between LMNA mutations and oxidative stress caused by excessive ROS accumulation is thought to be bidirectional: mutant forms of lamin A/C disrupt nuclear integrity increasing ROS production, and affected nuclei become more susceptible to oxidative stress (reviewed in^77^). Elevated ROS levels in fibroblasts and adipocytes have been proposed as an important factor contributing to the pathogenesis of laminopathies presented by FPLD2 and premature aging syndrome HGPS^10,11,77^. In our model hLMNA-R482L myoblasts and myotubes also accumulated ROS at greater rates compared to wildtype (Fig. 4d,e). In mutant myoblasts the increased ROS levels were associated with the activation of glutathione stress defense system (Fig. 5), although the mitochondrial respiration parameters were not altered (Fig. 7). Moderate ROS levels in skeletal muscles are known to play an important role in signal transduction and are increasing during myoblasts differentiation^13,17^. ROS should activate a metabolic regulator PGC-1α, and in this work we have shown the transcriptional activation of KEGG pathways PPAR signaling, cholesterol metabolism and fatty acid metabolism in mutant myoblasts (Fig. 4 a,b, Supplementary Table S3). Importantly, all these processes are associated with peroxisome functions, as well as ROS accumulation and scavenging, and hLMNA-R482L-induced disbalance in peroxisome-related mechanisms may be a cause of skeletal muscle abnormalities in FPLD2. Also, mutation-induced activation of autophagy (Fig. 4f–i) may contribute to the altered balance in cellular energy metabolism: autophagy consumes a large amount of ATP to provide substrates for protein biosynthesis, but also generates energy by degrading organelles, proteins, lipids^60^.

During the hLMNA-R482L myoblast-to-myotubes transition the increased ROS levels were not accompanied by the activation of antioxidant defense system (Fig. 5h). The unbalanced redox homeostasis and an accumulation of oxidative damage may contribute to defective skeletal muscle regeneration: C2C12 myoblasts with deletion of Gpx1 decrease the ability to fuse into myotubes in vitro despite the high levels of expression of factors stimulating muscle differentiation (reviewed in^78^). Additionally, we observed impaired cell respiration parameters in hLMNA-R482L myotubes, including high proton leak (Fig. 7) that might be both the cause and the sign of excessive ROS accumulation^79,80^.

It is known that the shift to glycolysis in developing/regenerating muscles provides the important advantages, including the ability not only rapidly generate ATP, but also the necessary intermediates for the biosynthesis of new macromolecules for biomass production via the pentose phosphate pathway (PPP)^16,81^. The downregulation of glycolysis pathway and PPP as well as the pathways that regulate the generation of the building blocks for the biosynthesis of amino acids and nucleotides in hLMNA-R482L myotubes (Fig. 6c–f) should contribute to both bioenergetic dysfunction and impaired differentiation causing muscular dystrophy. The inability of hLMNA-R482L cells to appropriately maintain the shift from glycolytic to OxPhos ATP production in course of differentiation (Fig. 7f,j) also supports the assumption that the metabolic dysfunction contributes to skeletal muscle abnormalities in FPLD2. Our findings are in a line with the work by Boschmann et al.^5^ who described skeletal muscle metabolism in patients with FPLD2 related to LMNA mutations (R482Q/R582H/R482W). Authors showed the shifts in the balance between lipid oxidation and oxidative glucose metabolism and speculated that insulin resistance in FPLD2 was related to alterations in insulin-mediated glucose oxidation rather than to insulin-mediated glucose uptake, the type of insulin resistance described by Randle et al.^82^. Same authors also reported that fatty acid oxidation in FPLD2 was largely incomplete and accompanied by increased ketogenesis, which is also in agreement with our data (Fig. 6a,b).

The other consequences of downregulation of both glycolysis and PPP pathway can be alterations in the balance between ROS accumulation and antioxidant defense glutathione system GSH/GSSG activation. Specifically, glucose in the form of glucose 6-phosphate (conversion downregulated in hLMNA-R482L, Fig. 6e), is the substrate for the first step in the PPP, which generates the electron carrier NADPH that donates electrons to antioxidant pathways (see the scheme on Fig. 5f), and the deficit of NADPH usually results in the decrease of efficiency of antioxidant defense systems. Consequently, we can speculate that the dysregulation in the coordination of molecular mechanisms that control cellular bioenergetics and redox homeostasis could be the cause not only in muscle tissue complications observed in patients with FPLD2, but also in other tissues of mesenchymal origin in patients with laminopathy^1–5,14^.

**Fig. 8:**
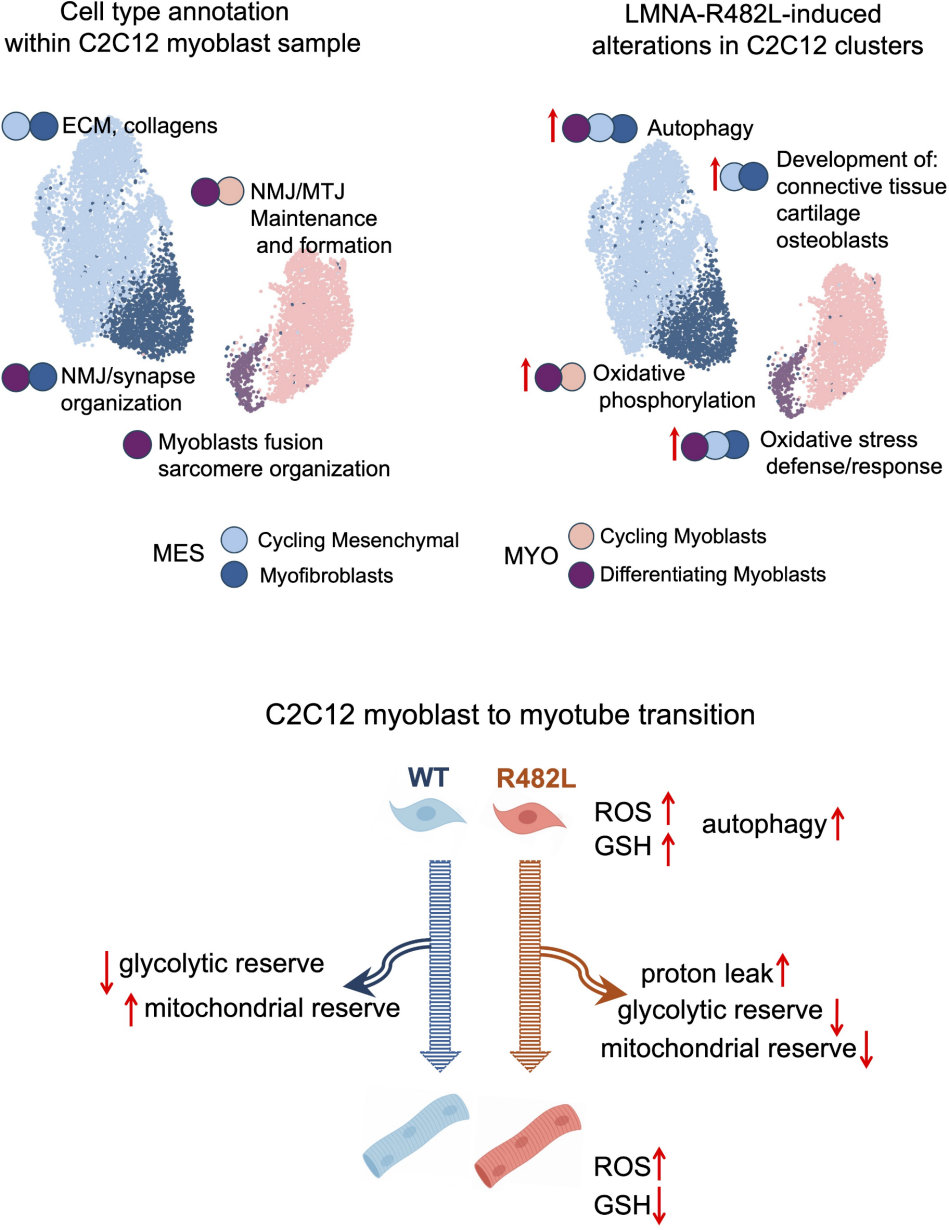
Summary for the results. C2C12 myoblast cluster annotation and LMNA-R482L induced changes in myoblasts and differentiating myotubes.

In summary, here we report transcriptional, redox-related and metabolic alterations in mouse myoblasts and myotubes bearing LMNA R482L mutation associated with FPLD2 (summarized in Fig. 8). We believe that these data extend our knowledge of the role of metabolic dysregulation and oxidative stress in the development of skeletal muscle complications in FPLD2 patients and provide important fundamental background for further investigation of molecular mechanisms of laminopathy pathogenesis.

In this project we employed the line of murine myoblasts genetically modified with lentiviral constructs to express human LMNA gene bearing R482L mutation associated with FPLD. As all the other cell-based in vitro models this approach has its limitations. First, there is endogenous expression of the native (WT) murine Lmna in both hLMNA-WT and hLMNA-R482L C2C12 samples, which can potentially affect the range of mutation-induced changes. Second, there is no proper environment in the samples of proliferating and differentiating in vitro C2C12 myoblasts; as a result, the cell-to-cell and tissue-cell interactions that contribute to skeletal muscle cells functioning in vivo are altered. Additionally, the lack of contractile activity in the differentiated in vitro myotubes affects its functionality and metabolism. Nevertheless, the model allowed for a detailed annotation of cell populations within the genetically modified C2C12 myoblast line, and an estimation of intrinsic mutation-induced functional and metabolic changes. These results will be used in future experiments with the C2C12 cell line to analyze dynamic changes in cellular populations in course of transition from cycling myoblasts to confluent myoblasts and differentiated myotubes providing additional information on mutation-associated changes that may affect myogenic differentiation and skeletal muscle function.

## Materials and methods

### Lentivirus production, infection, establishment of stable cell lines, cell culture and myogenic differentiation

The murine C2C12 (Catalog# ATCC CRL-1772) skeletal muscle cell line was purchased from the American Type Culture Collection (ATCC, Manassas, VA, USA). Undifferentiated, proliferating C2C12 cells were cultured in proliferation medium (DMEM supplemented with 4.5 g/L D-glucose, L-glutamine, penicillin–streptomycin, and 20% FCS). Differentiation was induced by replacing the proliferation medium with differentiation medium (DMEM media supplemented with 4.5 g/L D-glucose, L-glutamine, penicillin–streptomycin, and 2% Horse Serum). Differentiation medium was changed every day. Lamin A wild type and R482L mutant constructs, as well as establishment of stable cell lines of C2C12 hLMNA-R482L and hLMNA-WT myoblasts have been described in detail in our previous work^18^. The exact protocols are available upon request.

### Measurement of mitochondrial functions

Mitochondrial functions analyzed using the XFe24 flux analyzer and the mitochondrial stress test procedure as suggested by the manufacturer (Seahorse Bioscience): the culture medium was changed 60 min prior to the assay to Seahorse XF Base Medium supplemented with 1 mM of sodium pyruvate, 2.5 mM of glucose, and 1 mM of L-glutamine, pH was adjusted to 7.4. The oxygen consumption rate (OCR, pmol/min) measured in real time under basal conditions and after the addition of the following inhibitors: ATP synthase inhibitor oligomycin (2 μM); a mitochondrial protonophore uncoupler BAM15 (N5,N6-bis(2-fluorophenyl)[1,2,5]oxadiazolo[3,4-b]pyrazine-5,6-diamine) (10 μM); inhibitors of electron transport chain complexes I and III rotenone (1 μM), and antimycin A (1 μM) respectively^83^. The cells were then collected for determination of protein content (Pierce™ BCA Protein Assay Kit, Thermo Scientific™) for data normalization.

Metabolic parameters calculated based on OCR values for each well using Wave Desktop software. In order to have an opportunity to compare results from different experiments we used normalized data for calculations as recommended by manufacturer (User Guide for Kit 103015-100): spare respiratory capacity expressed as a fraction (%) of basal respiration = (maximal respiration) / (basal respiration) × 100; coupling efficiency calculated as = (ATP production) / (basal respiration) × 100; proton leak calculated as a fraction of basal respiration (%) = [((minimal OCR before oligomycin injection) – (minimal OCR after oligomycin injection) – (non-mitochondrial OCR))/(basal respiration)] × 100. Results analyzed using GraphPad Prism version 8.4.1 for Windows (GraphPad Software, USA).

### Real-time ATP rate assay

Agilent Seahorse XF Real-Time ATP Rate Assay was performed according to the manufacturer’s instructions (Seahorse Bioscience). 20k cells were seeded in each of 10 wells of cell culture plate (5 wells of LMNA-WT C2C12 and 5 wells of LMNA-R482L C2C12 myoblasts; 10 other wells were filled with cell culture medium until new portion of myoblasts seeded) and cultured for 24 h in proliferation medium. Then the proliferation medium was replaced with a differentiation medium and cultured for another 48 h (or 4 days depending on experimental design). Then, 20k cells were seeded into each of 10 empty wells (again, 5 wells of LMNA-WT C2C12 and 5 wells of LMNA-R482L C2C12 myoblasts). After 24h the OCR and ECAR were measured: cells were incubated with Seahorse XF DMEM Medium, pH 7.4 with 10 mM glucose, 1 mM sodium pyruvate, and 2 mM glutamine for an hour at 37 °C in a non-CO2 incubator. Before starting the XF assay, the medium was replaced with a fresh assay medium. OCR and ECAR were measured before and after the treatment with oligomycin (1.5 µmol), followed by rotenone/antimycin A (0.5 µmol each). Rates of oxygen consumption and extracellular acidification were expressed relative to the protein content of the appropriate well. Following metabolic parameters were calculated for each well using Wave 2.6.3 software: mitoATP (mitochondria-derived ATP) Production Rate = [(last OCR rate measurement before first oligomycin injection—minimum OCR rate measurement after oligomycin but before Rot/AA injection) × 2 x (P/O)]; glycoATP (glycolysis-derived ATP) Production Rate = last glycoPER measurement before first oligomycin injection; total ATP Production Rate = (mitoATP Production Rate) + (glycoATP Production Rate); Glycolytic Reserve Capacity = basal ECAR measurement—ECAR measurement after oligomycin injection. The Mann–Whitney statistical test was used.

### Reactive oxygen species measurement

For reactive oxygen species (ROS) detection, the fluorogenic ROS indicator 2′,7′-Dichlorofluorescein diacetate (DCF-DA, D6883, Sigma) was utilized. The cell-permeable nonfluorescent probe DCF-DA is oxidized to fluorescent DCF (2′,7′-Dichlorofluorescein) by a process involving ROS (reactive oxygen species) and is widely used to measure intracellular oxidants. There are limitations associated with the DCF assay for intracellular ROS measurement^84^: DCF-DA does not react directly all ROS species, therefore only the initial assessment of cellular redox state was evaluated, without determining of ROS species specificity^85^. We assume that samples from control and experimental transgenic cell lines exhibit the compatible efficiency in DCF radical generation.

DCF-DA was added to cells in serum-free medium in final concentration 5 μM for 30 min at 37 °C, as described. Exposure of C2C12 cells to H2O2 was used as a positive control. After incubation with a fluorogenic dye, the staining medium was removed and replaced with Gibco™ FluoroBrite™ DMEM. The fluorescence intensity was measured in a multi-mode plate reader CLARIOStar Plus (BMG LABTECH) using λex 485 nm and λem 530 nm. The assays were conducted with cells in Corning® 96-well Flat Clear Bottom Black Polystyrene TC-treated Microplates (REF3603). The Mann–Whitney statistical test was used.

### Relative quantification of mtDNA content using real-time PCR

Relative mitochondrial DNA (mtDNA) content was analyzed by Real-time PCR amplification of mtDNA-coded murine mitochondrial fragment and single nuclear reference gene Kcnj13 (inward rectifier potassium channel 13) using total DNA. Primer sequences are musMT553F23 (5′-GCCAGAGAACTACTAGCCATAGC-3′) and musMT668R23 (5′-AGCAAGAGATGGTGAGGTAGAGC-3′) for murine mitochondrial fragment; mus4987F25 (5′-GGATGAGAGAGAGAAGCACAAGTGG-3′) and mus5140R25 (5′-CTGTATGACCAACCTTGGACATGAT-3′) for Kcnj13^86^.

A quantitative real-time PCR assay was performed on an Applied Biosystems® 7500 Real-Time PCR system (Life Technologies, USA). Relative mtDNA content was determined by comparative Ct method using the equations:

$$\Delta {\text{Ct }} = {\text{ Ct nDNA }} - {\text{ Ct mtDNA}}$$

$${\text{Relative mitochondrial DNA content }} = { 2 } \times { 2}^{{\Delta {\text{Ct}}}}$$

Samples measured in qPCR represent the average of four biological replicates, with experiments repeated in two independent cell cultures for each cell line. The Mann–Whitney statistical test was used.

### Glutathione level measurement

Glutathione (GSH) levels in the living C2C12 myoblasts determined using Image ExFluorer system, and FreSHtracer, the reversible GSH probe that changes the absorption and fluorescence spectra when binds with GSH: in the presence of GSH, FreSHtracer fluorescence emission intensity at 580 nm decrease while increase at 510 nm, which allows the monitoring and comparison of cellular GSH levels based on fluorescence shifts. Cells were seeded on 12-well culture plates, cultured up to 50% density, incubated with FreSHtracer for 1.5 h, then the fluorescence ratio (F510/F580) was measured for each cell using Image ExFluorer system. Results analyzed using GraphPad Prism version 8.4.1 for Windows (GraphPad Software, USA). The Mann–Whitney statistical test was used for value comparison.

### LC3-I and LC3-II samples preparation for Western blot analysis

The analysis of autophagy flux using Western Blotting (WB) was based on the fact that the number of autophagosomes correlates with the amount of the LC3-II protein, which is specifically associated with autophagosomal membranes as described previously^87,88^. For this analysis, the membrane-unbound (cytosolic) LC3-I form was extracted from cells after permeabilization of cell membrane with digitonin, then the extraction of membrane-bound LC3-II fraction of LC3 protein was performed. Thus, the abundance of LC3-I and LC3-II isoforms in the samples estimated separately.

Briefly, 100k of C2C12 myoblasts were seeded on 6-well culture plates and cultured in differentiation medium for 24–48 h up to subconfluent condition. Then, to prevent the autophagosome degradation, 100 μM of autophagic flux inhibitor chloroquine (CQ) was added for 2 h to the experimental samples. Control samples were incubated for 2 h with the solvent only. The samples of LC3-I and LC3-II proteins were prepared as follows: cells were washed with PBS, trypsinized, collected into a sample tube, and centrifuged at 1000 g for 5 min. The pellet was placed on ice and incubated for 5 min in PBS with 0.025% of digitonin (Sigma, USA) for permeabilization. After centrifugation at 2000 g for 5 min, supernatant was collected for further analysis of the membrane-unbound cytosolic isoform LC3-I. Extraction of the membrane-bound LC3-II protein from the pellet was performed by incubation for 10 min in ice with RIPA buffer containing a cocktail of proteases inhibitors (Roche, USA), 1% NP-40, 0.5% sodium deoxycholate, 1% SDS, 1% Triton-X 100, 5 mM EDTA. The lysates were centrifuged at 16,000 g for 10 min, and the supernatant was collected for the analysis of membrane-bound LC3-II isoform. Laemmli buffer was added to all protein lysates and incubated for 5 min at 90 °C prior WB analysis.

### Western blot analysis

Extracts were separated by sodium dodecyl sulfate–polyacrylamide gel electrophoresis (SDS-PAGE) and transferred to 0.45 µm pore size nitrocellulose membrane (1620115, Bio-Rad, Hercules, CA, USA). LC3-I and LC3-II isoforms analyzed separately on individual gels. Western blot was performed using standard procedures, with blocking in 5% skimmed milk for 1 h and washes in PBS containing 0.05% Tween 20. Membranes were probed at 4 °C overnight with target antibody: anti-LC3 polyclonal rabbit antibody (MBL International Corporation, USA); anti-Desmin monoclonal mouse antibody (DAKO); monoclonal mouse anti-aSMA antibody (Novus Biologicals); Phalloidin Red (Invitrogen); Vinculin (Invitrogen).

The blots were revealed by secondary HPR-conjugated antibodies (#1706516, Bio-Rad, Hercules, CA, USA), and SuperSignal West Femto substrate (Thermo Fisher Scientific). Chemiluminescence was detected using the Fusion Fix imaging system (Vilber Lourmat, Marne La Vallee, France), and analyzed with FusionCapt Advance FX7; the Mann–Whitney statistical test was used for value comparison.

### Immunocytochemistry

LC3-II staining: cells were grown up on coverslips up to subconfluent density. Then, 100 μM of autophagic flux inhibitor chloroquine (CQ) was added for 2 h followed by permeabilization with 0.005% digitonin in PBS for 5 min on ice. Then cells washed 3 times with PBS for 5 min to remove the unbound LC3-I fraction. After that cells were fixed with 4% paraformaldehyde for 5 min on ice and blocked with 15% FCS in PBS at RT for 30 min. Incubation with the primary polyclonal anti-LC3 polyclonal antibody (MBL International Corporation, USA) diluted in PBS 1:500 was performed at RT for 1 h. Goat anti-rabbit secondary antibody (Alexa Fluor 560, Thermo Fisher Scientific, USA) diluted 1:200 in PBS applied for 45 min at RT in the dark. DAPI (Invitrogen, USA) was used to counterstain nuclei.

Desmin/F-actin/aSMA staining: cells were grown on cover glasses, fixed in 4% paraformaldehyde for 10 min at 4 °C (myoblasts), permeabilized with 0.02% Triton X-100 for 5 min. To block the nonspecific binding incubated for 30 min with 15% FCS. Then 1 h incubation with primary mouse monoclonal (D33, DAKO) antibodies, aSMA (Novus Biologicals) antibodies and Phalloidin Red (Invitrogen) performed followed by 45 min incubation with the secondary antibodies conjugated with Alexa Fluor 546/Alexa-488 (Molecular Probes, Eugene, OR, USA). Nuclei were counterstained with DAPI (Molecular Probes, USA).

### RNA-seq data analysis

Gene Expression Omnibus (GEO) dataset GSE150365 from our previous study^18^ was used for the detailed analysis of bulk RNA-seq of C2C12 transgenic hLMNA-WT/hLMNA-R482L myoblasts in the process of differentiation. Briefly, reads were aligned to the mouse genome GRCm38 vM22 using STAR^89^ (v2.6.1a), read count table was generated with featureCounts^90^ (v1.6.2). Differential expression (DE) analysis was performed with R (v4.2.2) using DESeq2^91^ (v1.38.3) package with filtering parameters abs (fold change) > 1.5 and False Discovery Rate (FDR) = 0.05 for DE genes. For the identification of the de-regulated genes in the process of differentiation comparing to control WT cells, likelihood ratio test (LRT) was applied on DESeq-normalized counts (FDR = 0.01); gene clusters were extracted with degPatterns function from DEGreport package (v1.34.0)^92^. Pathway analysis on gene sets and ordered vector of genes (Gene Set Enrichment Analysis) was performed with clusterProfiler^93^ (v4.6.2) and fgsea^94^ (1.24.0) R packages using Gene Ontology (GO) biological process (2022-07-01) and Kyoto Encyclopedia of Genes and Genomes (KEGG, 2023) pathway datasets. Dysregulated metabolic modules were found using GATOM web-application^61^ that uses DE genes to find possibly de-regulated metabolites and modules with FDR = 0.05.

### Single-cell RNA-seq library preparation and sequencing

Single-cell libraries construction was performed with the Chromium Controller and the Single Cell 3′ Reagent Kit v3.1 (10 × Genomics) according to the manufacturer’s protocol. Briefly, C2C12 myoblasts were grown up to subconfluence, trypsinized, washed 2 × with PBS. Cell suspensions with the targeted cell recovery rate 5000 cells per sample were loaded onto 10 × Genomics Single Cell fluidics chips to generate GEMs (Gel Beads-in-emulsion) which include gel beads coated with specific oligonucleotide barcodes to index each cell’s transcriptome and Unique Molecular Identifiers (UMI) to define individual transcripts. Uniquely barcoded RNAs were reverse transcribed, and individual cDNAs were amplified using a bench top Veriti™ 96-well thermal cycler (Applied Biosystems). Each library was barcoded with different indexes from a Chromium i7 Multiplex Kit. Barcoded libraries were then pooled and sequenced on the Illumina NextSeq 2000 system. Sequenced libraries are presented with 1 sample of hLMNA-WT and 2 samples of hLMNA-R482L C2C12 myoblasts. FASTQ files and count matrixes are available at NCBI Gene Expression Omnibus database under the accession number GSE260946.

### Single cell RNA-seq data analysis

Raw reads were processed using Cell Ranger v6.1.2 software aligning to the mouse genome (Cell Ranger mouse reference 2020-A, based on GRCm38/GENCODE vM23). Filtered count matrixes from Cell Ranger were processed using Seurat (v4.3.0) R package^95^; cells with mitochondrial content > 15% and UMI < 500 were removed from the analysis. In average 120 million reads and 3200 cells for each sample were produced with median unique molecular identifier (UMI) counts per cell 15,087 and median genes per cell 4262; in total 9470 cells (3149 for hLMNA-WT and 6321 for hLMNA-R482L) were taken for further analysis. Samples were transformed using SCTransform function regressing out mitochondrial content and cell cycle genes, and integrated. Unsupervised graph-based cell clustering with resolution parameter 0.15 revealed 4 cell subpopulations. Cluster markers and differentially expressed genes between the conditions WT/R482L were obtained using FindConservedMarkers and FindMarkers functions from Seurat correspondingly (FDR = 0.01) and analyzed with clusterProfiler R package for the pathway identification (FDR = 0.05). Pseudotemporal ordering of cells was assessed using monocle3 (v1.3.1)^96^. Violin plots were drawn to visualize the distribution of bulk RNA-seq GSEA pathways among single cell RNA-seq clusters: the percentage of counts for upregulated bulk RNA-seq DEGs from these pathways for each cell was calculated; values were than compared between WT and R482L C2C12 cells for each cluster using Wilcoxon rank sum test.

## Supplementary Information

Supplementary Information 1.

Supplementary Information 2.

Supplementary Information 3.

Supplementary Information 4.

Supplementary Information 5.

## Data Availability

The single-cell RNA-seq dataset generated during the current study is available in the NCBI Gene Expression Omnibus repository under the accession number GSE260946, https://www.ncbi.nlm.nih.gov/geo/query/acc.cgi?acc=GSE260946. The bulk RNA-seq dataset analyzed in this study is available in the NCBI Gene Expression Omnibus repository under the accession number GSE150365, https://www.ncbi.nlm.nih.gov/geo/query/acc.cgi?acc=GSE150365.
