# Supplementary material for: LMNA R482L mutation causes impairments in C2C12 myoblasts subpopulations, alterations in metabolic reprogramming during differentiation, and oxidative stress
Source: Sci Rep. 2025 Feb 13;15:5358. doi: 10.1038/s41598-025-88219-6 (PMC11825939; doi:10.1038/s41598-025-88219-6)

Supplementary Figures for the article “LMNA R482L mutation causes impairments in C2C12 myoblasts subpopulations, alterations in metabolic reprogramming during differentiation, and oxidative stress” by Ivanova O. et.al.

Figure S1. Single cell RNA-seq cluster distribution for 3 samples of hLMNA-R482L/WT C2C12 myoblasts. A) Proportion of cells for all 4 C2C12 clusters and b) for two populations MES and MYO separately. C) UMAP plot for 3 samples of hLMNA-R482L/WT C2C12 myoblasts.

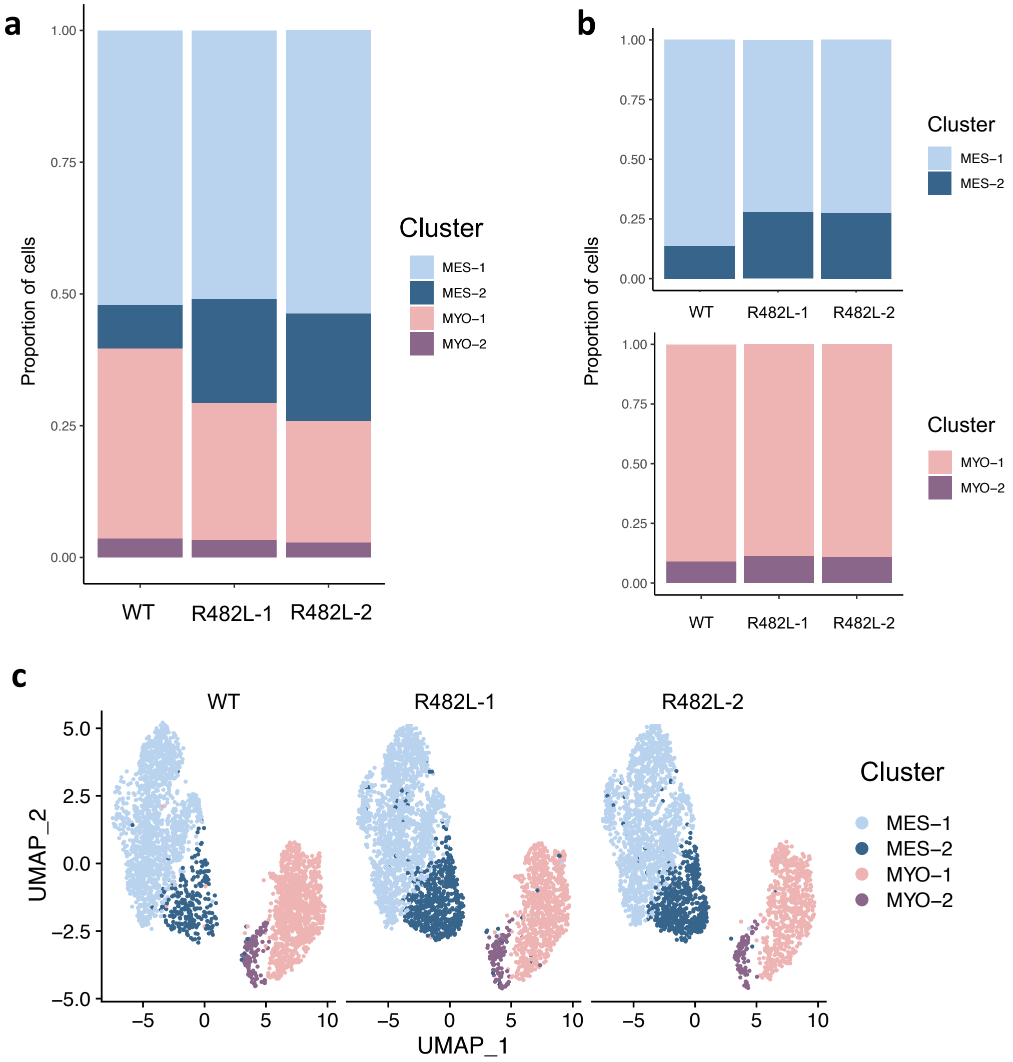

Figure S2. Western blotting original files for Figures 2i and 4g. Western blotting was performed for C2C12 myoblasts bearing LMNA-R482L and LMNA-WT genes as described in Methods.

Western Blotting Original Files for Figure 2i

aSMA

Loading control:
Vinculin

Desmin

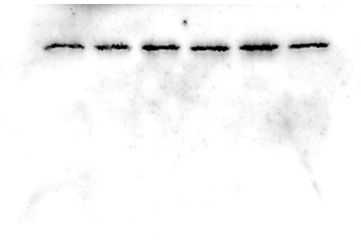

Loading control:
Vinculin

Western Blotting Original Files for Figure 4g

LC3-I (cytosol)

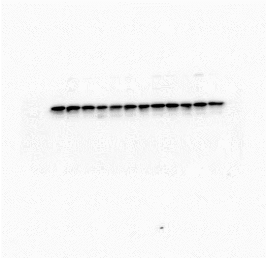

LC3-II (membrane-bound)

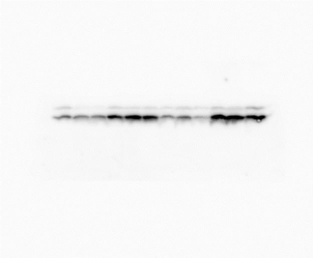

Loading control for LC3-I:
(cytosol)

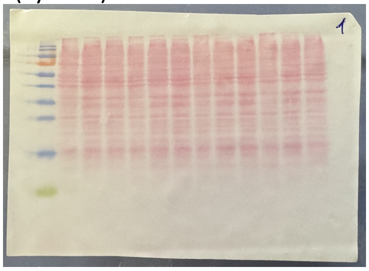

Loading control for LC3-II:
(membrane-bound)

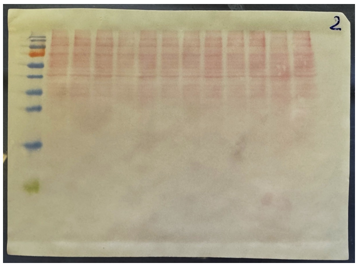

Figure S3. Results of bulk RNA-seq differential expression analysis of hLMNA-R482L/WT C2C12 myoblasts.
(a) Volcano plot shows the DEGs in the R482L/WT C2C12 myoblasts (abs (Fold Change) > 1.5, FDR=0.05); the full table of DEGs and corresponding pathways is in Supplementary Table S3.
(b) Graph of the interconnections between pathways and corresponding core enriched genes taken from GSEA results (Supplementary Table S3).

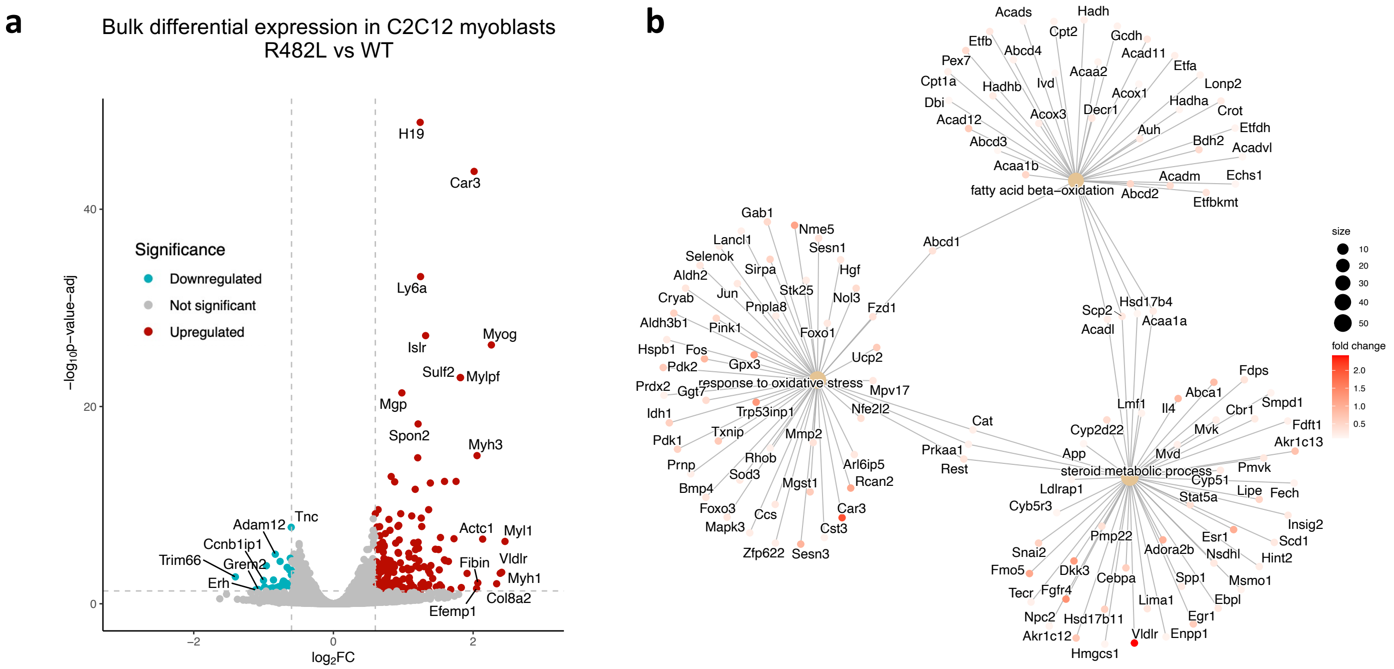

Figure S4. The results of Likelihood Ratio Test (LRT) from DEGreport R package assessing significantly de-regulated gene clusters in course of myogenic differentiation (days 0, 2, 5) of hLMNA-R482L/WT C2C12 myoblasts from RNA-seq dataset GSE150365. List of genes is available in Supplementary Table S4.

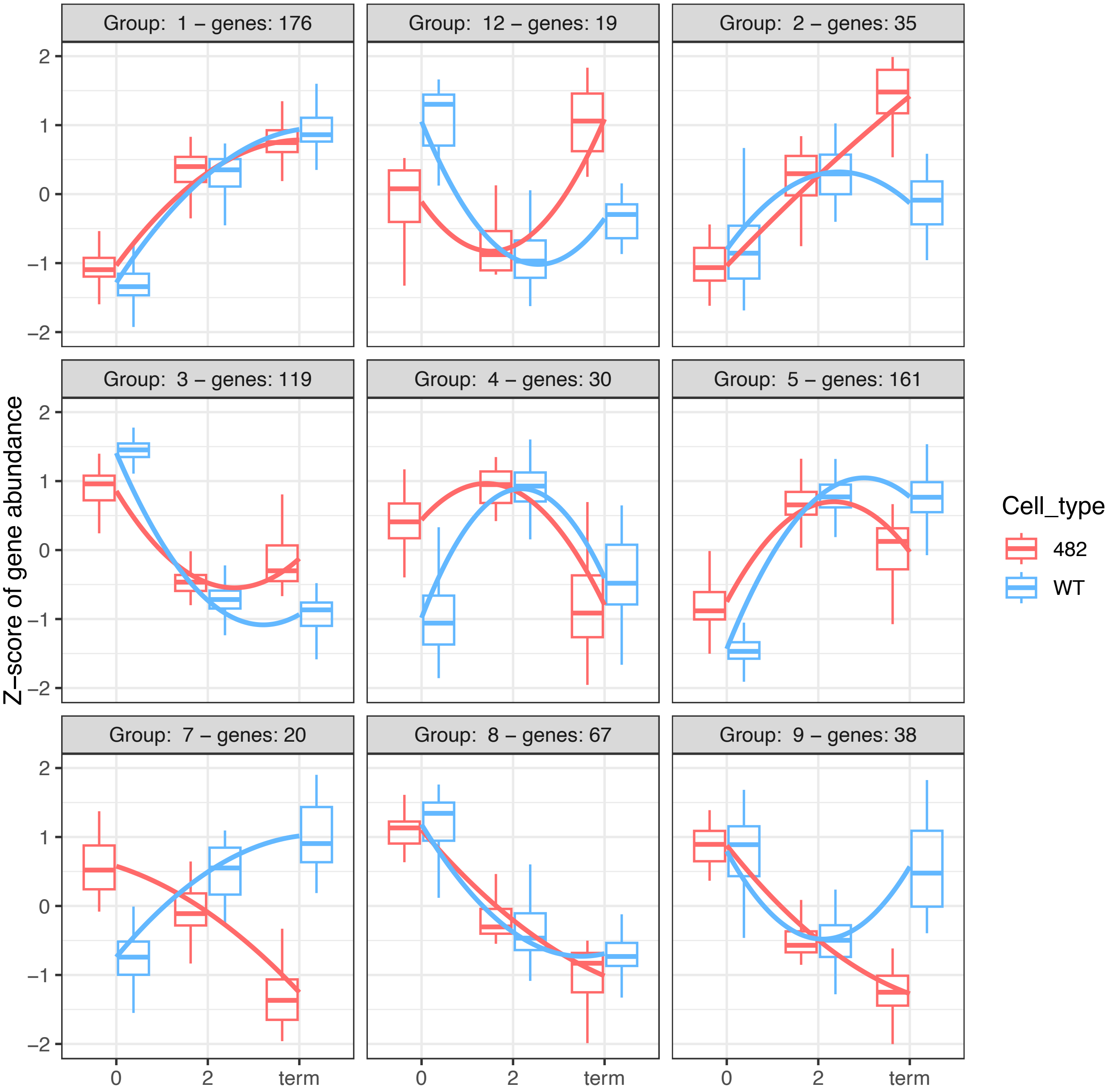

Supplement: Supplementary file 1 — Supplementary Information 1. [file 41598_2025_88219_MOESM1_ESM.pdf]
